# Cannabinoid-Driven Rewiring of GPCR and Ion Channel Signaling in Lung Cancer

**DOI:** 10.3390/biomedicines14040856

**Published:** 2026-04-09

**Authors:** Didik Setyo Heriyanto, Fahrul Nurkolis, Jinwon Choi, Sohyun Park, Min Choi, Raymond Rubianto Tjandrawinata, Amama Rani, Moon Nyeo Park, Min-Jin Kwak, Bum Sang Shim, Bonglee Kim

**Affiliations:** 1 Department of Anatomical Pathology, Faculty of Medicine, Public Health and Nursing, Universitas Gadjah Mada/Dr. Sardjito, General Hospital, Yogyakarta 55284, Indonesia; 2 Division of Cardiac, Thoracic, and Vascular Surgery, Department of Surgery, Faculty of Medicine, Public Health, and Nursing, Universitas Gadjah Mada/Dr. Sardjito, General Hospital, Yogyakarta 55284, Indonesia; 3 Collaboration Research Center for Precision Oncology Based Omics-PKR PrOmics, Faculty of Medicine, Public Health, and Nursing, Universitas Gadjah Mada, Yogyakarta 55281, Indonesia; 4 Faculty of Medicine, Universitas Airlangga, Surabaya 60131, Indonesia; fahrul.nurkolis.mail@gmail.com; 5 Medical Research Center of Indonesia, Surabaya 60281, Indonesia; 6 Institute for Research and Community Service, State Islamic University of Sunan Kalijaga (UIN Sunan Kalijaga), Yogyakarta 55281, Indonesia; 7 Department of Pathology, College of Korean Medicine, Kyung Hee University, Seoul 02447, Republic of Korea; 8 School of Bioscience, Innovation and Technology, Atma Jaya Catholic University of Indonesia, Jakarta 12930, Indonesia; 9 Department of Microbiology, Quaid-i-Azam University, Islamabad 45320, Pakistan; 10 Department of Forest Products and Biotechnology, Kookmin University, Seoul 02707, Republic of Korea; 11 Korean Medicine-Based Drug Repositioning Cancer Research Center, College of Korean Medicine, Kyung Hee University, Seoul 02447, Republic of Korea

**Keywords:** lung cancer, cannabinoids, GPCR signaling, ion channels, CB1 receptor, CB2 receptor, TRP channels, signaling rewiring, therapy resistance, targeted therapy

## Abstract

Lung cancer remains the leading cause of cancer-related mortality worldwide, with non-small cell lung cancer accounting for the majority of cases and exhibiting persistent challenges related to therapy resistance and metastatic progression. Increasing evidence indicates that dysregulated G protein-coupled receptor signaling and ion channel activity function cooperatively as master regulators of tumor cell proliferation, migration, survival, and therapeutic response. Cannabinoids, including phytocannabinoids such as delta-9-tetrahydrocannabinol and cannabidiol, as well as endogenous endocannabinoids, are uniquely positioned to modulate both G protein-coupled receptors and ion channels, thereby influencing key oncogenic signaling networks. This review synthesizes current knowledge on the role of major ion channel families, including transient receptor potential channels, potassium channels, and sodium channels, and principal G protein-coupled receptor pathways involved in lung cancer progression. We further discuss how cannabinoids reprogram these interconnected signaling systems through canonical cannabinoid receptors, non-classical targets such as G protein-coupled receptor 55 and adenosine receptors, and direct modulation of ion channel activity. Special attention is given to G protein-coupled receptor–ion channel coupling within membrane microdomains and to the capacity of cannabinoids to act as biased ligands, redirecting downstream pathways, such as the phosphoinositide 3-kinase–protein kinase B–mechanistic target of rapamycin and epidermal growth factor receptor signaling, toward apoptosis and reduced metastatic potential. Emerging strategies, including cannabinoid-based combination therapies, selective receptor biasing, and targeted delivery systems, are also highlighted. Altogether, cannabinoid-driven rewiring of G protein-coupled receptor and ion channel signaling represents a promising mechanistic framework for developing innovative therapeutic approaches against lung cancer.

## 1. Introduction

Lung cancer remains the leading cause of cancer-related mortality worldwide, with non-small cell lung cancer (NSCLC) accounting for ~85% of cases [1]. Despite major advances in targeted therapies (e.g., EGFR and ALK inhibitors) and immunotherapies, long-term survival remains poor due to treatment resistance and metastatic progression [2,3]. This limitation has spurred interest in non-classical therapeutic targets, particularly G-protein-coupled receptors (GPCRs) and ion channels, which function as master regulators of cell signaling and tumor biology. GPCRs form a vast receptor family that activates heterotrimeric G-proteins (Gα and Gβγ subunits), controlling second messengers like cyclic AMP (cAMP), Ca^2+^, lipid mediators, and kinase cascades. In parallel, ion channels regulate ionic homeostasis and membrane potential, thereby modulating proliferation, apoptosis, migration, and angiogenesis in cancer cells [4,5]. An emerging paradigm is that aberrant GPCR and ion channel activities cooperate to sustain oncogenic signaling in lung cancer, representing a “signaling network” ripe for therapeutic intervention.

Cannabinoids are a class of compounds that include the phytocannabinoids Δ^9^-tetrahydrocannabinol (THC) and cannabidiol (CBD), as well as endogenous endocannabinoids [6]. These are unique because they engage both GPCRs and ion channels. The canonical cannabinoid receptors, CB1 and CB2 (encoded by *CNR1* and *CNR2*), are GPCRs abundantly expressed in the nervous and immune systems, respectively [7,8]. Notably, both CB1 and CB2 are expressed in lung tumors [7,9], and their activation by exogenous cannabinoids can modulate cancer cell fate. Indeed, preclinical studies show that THC and CBD reduce NSCLC cell proliferation, migration, and epithelial–mesenchymal transition (EMT) markers [9], indicating anti-tumor effects via these receptors. Beyond CB1/CB2, other GPCRs influence lung cancer and can be targeted or modulated by cannabinoids: for example, the orphan receptor GPR55 (sometimes termed a non-classical cannabinoid receptor) and the adenosine receptors (A2A, A2B, and A3) are implicated in tumor growth, metastasis, and immune evasion. In parallel, a variety of ion channels are dysregulated in lung cancer, notably Ca^2+^-permeable transient receptor potential (TRP) channels, voltage-gated K^+^ channels, Ca^2+^-activated K^+^ channels, and voltage-gated Na^+^ channels, endowing cancer cells with specialized “electrophysiological” phenotypes that promote malignant behavior. Intriguingly, cannabinoids can directly and indirectly modulate several of these channels. For instance, the endocannabinoid anandamide (AEA) is an agonist of the Ca^2+^-permeable TRPV1 channel, and CBD is known to activate TRPV2 channels [7,10]. Thus, cannabinoids occupy a unique position as electrophysiological modulators that can rewire signaling at the intersection of GPCR and ion channel pathways.

This review synthesizes current knowledge on the interplay between GPCR signaling and ion channel activity in lung cancer and examines how cannabinoids modulate these interconnected pathways. By integrating electrophysiological regulation with receptor-mediated signaling, this work highlights a unified framework through which cannabinoids influence tumor cell behavior and identifies emerging opportunities for therapeutic intervention.

## 2. Ion Channels in Lung Cancer: Master Regulators of Oncogenic Signaling

**Figure 1:**
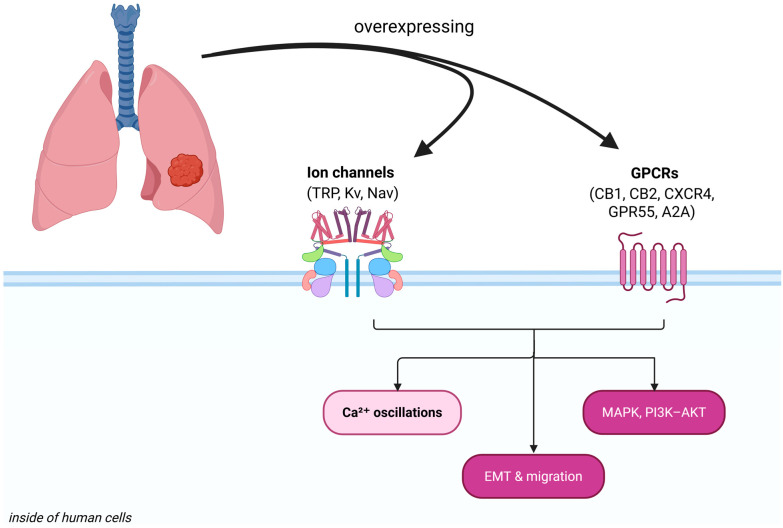
Aberrant GPCR and ion channel signaling networks driving lung cancer progression. Dysregulated GPCR activation and ion channel activity converge to control intracellular Ca^2+^ dynamics, membrane potential, and migratory behavior, thereby promoting proliferation, epithelial–mesenchymal transition (EMT), and therapy resistance in lung cancer cells. Color differences are used to distinguish signaling layers, where Ca^2+^ oscillations represent early second messenger dynamics (light shading), while MAPK, PI3K–AKT, and EMT/migration represent downstream oncogenic signaling and phenotypic outputs (dark shading). This figure was created via Biorender Premium License by Fahrul Nurkolis.

Ion channels have increasingly been recognized as important contributors to the hallmarks of cancer, even in non-excitable cells such as lung epithelial cells [4]. By controlling transmembrane fluxes of Ca^2+^, K^+^, Na^+^, and other ions, these channels regulate proliferation, apoptosis, cell volume, motility, and the interaction between cancer cells and their microenvironment. Lung cancer cells often co-opt ion channels to create a pro-tumorigenic ionic milieu: For example, elevated intracellular Ca^2+^ can drive proliferation and invasion, while K^+^ efflux can hyperpolarize the membrane to facilitate Ca^2+^ entry and maintain a permissive voltage for cell cycle progression [11]. Below, we discuss three major classes of ion channels, such as calcium-permeable TRP channels, potassium channels, and sodium channels, that are frequently dysregulated in lung cancer. Figure 1 illustrates these channels and their roles in lung cancer cells. Table 1 provides a summary of their characteristics and oncogenic functions.

### 2.1. Calcium-Permeable Channels: TRP Channels and Calcium Influx

Transient receptor potential (TRP) channels form a large family of non-selective cation channels that often allow Ca^2+^ influx. Several TRP subtypes have been implicated in lung cancer, including the vanilloid subfamily (TRPV1 and TRPV2), canonical subfamily (TRPC1), and melastatin subfamily (TRPM7).

#### 2.1.1. TRPV1 (Transient Receptor Potential Vanilloid 1)

Best known as the capsaicin receptor in sensory neurons, TRPV1 is abundantly expressed in some lung tumors. Recent studies show that *TRPV1 mRNA* and proteins are significantly upregulated in NSCLC tissues, with higher TRPV1 correlating with poor patient survival [12,13]. Functionally, TRPV1 appears to act as a tumor promoter in NSCLC. Wang et al. (2024) [14] demonstrated that TRPV1 knockdown in NSCLC cells inhibited proliferation and induced apoptosis via suppression of a Ca^2+^-IGF1R (insulin-like growth factor 1 receptor) signaling axis. Loss of TRPV1 also shifted the tumor microenvironment toward an immune-reactive state, increasing cytotoxic T cell infiltration while reducing immunosuppressive myeloid cells [14]. Conversely, TRPV1 activation can support cancer cell survival: Li et al. (2022) found that overexpression of TRPV1 rendered NSCLC cells resistant to cisplatin and 5-fluorouracil by a mechanism independent of the channel’s acute Ca^2+^ flux function [3]. TRPV1-overexpressing cells upregulated the drug efflux transporter ABCA5 and enhanced DNA repair (homologous recombination), thereby evading chemotherapy-induced apoptosis. They also secreted more IL-8 (a pro-survival chemokine) upon TRPV1 overexpression. Clinically, TRPV1 overexpression has been associated with chemoresistance and shorter disease-free survival in lung cancer [3,15]. These findings paint TRPV1 as an oncogenic Ca^2+^ channel in lung cancer, promoting cell cycle progression, survival signaling (e.g., via IGF1R and IL-8), and therapy resistance. Targeting TRPV1 (with antagonists or gene silencing) may therefore have therapeutic benefits. Indeed, TRPV1 inhibition/knockdown significantly slowed NSCLC xenograft growth in vivo and enhanced anti-tumor immunity in one study [14]. Interestingly, while constitutive TRPV1 activity supports tumor growth, acute pharmacological activation of TRPV1 can be cytotoxic to cancer cells by Ca^2+^ overload. Capsaicin, a TRPV1 agonist, has shown pro-apoptotic effects in SCLC and other cancers, suggesting a complex role where persistent TRPV1 signaling aids tumor progression but strong activation can trigger cell death [16,17]. This dichotomy provides a rationale for exploring cannabinoid interactions with TRPV1, as discussed later, since certain endocannabinoids and CBD can activate or sensitize TRPV1 channels.

#### 2.1.2. TRPV2 (Transient Receptor Potential Vanilloid 2)

TRPV2 is another Ca^2+^-permeable channel that, in contrast to TRPV1, appears to *restrain* lung tumor aggressiveness under some conditions. TRPV2 expression has been noted in lung adenocarcinoma cells, and intriguingly, high TRPV2 levels correlate with better overall survival in lung cancer patients [10,18]. Mechanistically, TRPV2 can mediate Ca^2+^ influx that promotes apoptotic or autophagic cell death in cancer cells. A recent breakthrough by Misri et al. (2022) [10] demonstrated that CBD (cannabidiol) exerts anti-tumor effects in cisplatin-resistant lung cancer cells by activating TRPV2. CBD triggered TRPV2-dependent Ca^2+^ influx, leading to apoptosis of drug-resistant NSCLC cells and suppression of their growth and metastasis in vivo [10]. In TRPV2-expressing lung adenocarcinoma models, CBD treatment drove up reactive oxygen species (ROS) and downregulated NRF2 (a master regulator of antioxidant defenses), tipping the balance toward cell death. Notably, knocking down TRPV2 or pharmacologically blocking it abrogated CBD’s pro-apoptotic effect, confirming TRPV2 as a critical mediator. These findings designate TRPV2 as a tumor-suppressive channel when activated, especially in the context of overcoming chemoresistance. TRPV2 activation by CBD also reduced cancer stem cell properties and metastasis in mouse models [10,19]. Thus, TRPV2 stands out as a promising target to exploit, where cannabinoids could be used as TRPV2 agonists to selectively kill resilient cancer cells. Endogenous regulators of TRPV2 in tumors are not fully elucidated, but our evolving understanding suggests that reinstating TRPV2 activity in lung cancer (e.g., via CBD or analogues) may counteract malignant progression.

#### 2.1.3. TRPC1 (Transient Receptor Potential Canonical 1)

TRPC1 is a classical store-operated Ca^2+^ entry (SOCE) channel that opens in response to endoplasmic reticulum Ca^2+^ store depletion. Aberrant TRPC1 activity can chronically elevate cytosolic Ca^2+^ levels and activate Ca^2+^-dependent signaling in cancer cells. In NSCLC, TRPC1 is frequently overexpressed, and its high expression is associated with advanced tumor stage, lymph node metastasis, and shorter disease-free survival [20,21]. Immunohistochemical analyses of 192 NSCLC patient samples showed TRPC1 protein levels were significantly higher in tumors than in normal lung tissue, with 90% of cases staining positive. Patients with TRPC1-overexpressing tumors had worse DFS (and a trend toward worse overall survival) compared to those with low TRPC1 [20]. Functionally, TRPC1 enhances multiple malignant behaviors. It promotes NSCLC cell proliferation and cell-cycle progression: Silencing TRPC1 in lung carcinoma cell lines induced G0/G1 arrest and dramatically reduced growth. TRPC1 also facilitates cell migration and invasion, partly by enabling sustained Ca^2+^ influx that activates Ca^2+^-dependent proteases and motility pathways. An important crosstalk exists between TRPC1 and receptor tyrosine kinases in lung cancer. TRPC1 has been identified as a major regulator of epidermal growth factor receptor (EGFR) signaling in NSCLC cells [22]. Specifically, TRPC1 activity helps maintain basal AKT phosphorylation and EGFR-driven proliferation, likely by providing necessary Ca^2+^ for downstream signaling [22,23]. Under hypoxic conditions, TRPC1 was shown to mediate hypoxia-induced EGFR and STAT3 activation in lung cancer cells, linking microenvironmental stress to aggressive signaling [23]. These observations place TRPC1 at a nodal point connecting Ca^2+^ dynamics with oncogenic kinase pathways. From a therapeutic standpoint, TRPC1 could be targeted to disrupt this coupling: Small-molecule TRPC inhibitors (e.g., SKF-96365, 2-APB) have been reported to cause cell cycle arrest and growth inhibition in lung cancer cells. There is also evidence that certain compounds (carvacrol, capsaicin) can inhibit lung cancer cell viability by affecting TRP channels, including TRPCs [16,24]. Overall, TRPC1 acts as an oncogenic Ca^2+^ conduit in lung cancer, driving proliferation, invasion, and metastasis. Its overexpression is associated with poor prognosis in NSCLC, and it functionally couples GPCR-mediated signaling pathways to TRPC1-dependent Ca^2+^ entry via PLCβ and IP_3_.

#### 2.1.4. TRPM7 (Transient Receptor Potential Melastatin 7)

TRPM7 is a unique bifunctional protein with both ion channel and serine/threonine kinase domains (a “chanzyme”). It conducts Ca^2+^ and Mg^2^ and is vital for cellular homeostasis. TRPM7 is aberrantly expressed in many malignancies, including lung cancer. High *TRPM7* expression in lung tumors has been linked to enhanced tumor cell motility, metastatic potential, and poorer prognosis [4,25]. One analysis found TRPM7 upregulated in lung cancer tissues and cell lines and identified TRPM7 as an independent predictor of worse overall survival in lung cancer patients. Functionally, TRPM7 promotes a more aggressive, stem-like phenotype. Its activity has been shown to sustain the cancer stem cell (CSC) subpopulation in lung cancer, contributing to chemoresistance and recurrence. TRPM7′s kinase domain can modulate signaling pathways such as Notch and STAT3, which are known to maintain stemness, as seen in glioma models [4,26]. In lung cancer cells, TRPM7 supports epithelial–mesenchymal transition (EMT) and metastasis by controlling downstream effectors like Hsp90α, uPA, and MMP-2 (matrix metalloproteinase-2) [27]. In vitro, silencing TRPM7 or blocking it (e.g., with waixenicin A, a TRPM7 inhibitor) reduced lung cancer cell migration/invasion and sensitized cells to chemotherapy [4]. TRPM7 may also interface with metabolic reprogramming in cancer: Recent work indicates TRPM7 activity can drive aerobic glycolysis (the Warburg effect) to fuel tumor growth [28]. Additionally, growth factor signals can influence TRPM7; for example, EGF stimulation was reported to upregulate TRPM7 currents and enhance A549 cell migration, whereas TRPM7 knockdown abrogated EGF-induced motility [29]. Thus, TRPM7 links external signals to internal Ca^2+^ and Mg^2^ dynamics, fostering a pro-tumor state. Therapeutically, targeting TRPM7 is challenging due to its importance in normal physiology, but it remains an attractive target given its multifaceted role in proliferation, CSC maintenance, and metastasis. Efforts to develop TRPM7-selective inhibitors or exploit synthetic lethality (targeting its kinase vs. channel function) are ongoing [30,31]. In summary, TRPM7 is an oncogenic ion channel in lung cancer that drives motility, invasion, and therapy resistance, making it a critical component of the lung tumor ionic signaling network.

**Table 1:** Major ion channels implicated in lung cancer. Summary of key ion channels, their ion selectivity, and oncogenic roles in lung cancer.

Ion Channel	Type/Ion Selectivity	Expression & Role in Lung Cancer	Representative References
TRPV1 (Transient Receptor Potential Vanilloid 1)	Non-selective cation channel (Ca^2+^, Na^+^) activated by heat, capsaicin, low pH	↑ in NSCLC; correlates with poor prognosis Promotes proliferation & survival (Ca^2+^–IGF1R signaling) Mediates chemoresistance (↑ DNA repair, drug efflux) Knockdown → ↓ tumor growth, ↑ anti-tumor immunity	[3,14]
TRPV2 (Transient Receptor Potential Vanilloid 2)	Non-selective cation channel (Ca^2+^, Na^+^) activated by heat, CBD, etc.	Expressed in lung adenocarcinoma; associated with improved survival Activation (e.g., cannabidiol) → apoptosis in resistant cells Tumor-suppressive role via Ca^2+^ influx → ROS generation Overcomes chemoresistance	[10]
TRPC1 (Transient Receptor Potential Canonical 1)	Non-selective cation channel (Ca^2+^ entry store-operated)	Overexpressed in NSCLC; linked to metastasis & advanced stage Drives proliferation & migration (sustained Ca^2+^ entry) Enhances EGFR and AKT signaling Predicts shorter disease-free survival	[20,22]
TRPM7 (Transient Receptor Potential Melastatin 7)	Non-selective cation channel (Ca^2+^, Mg^2+^); contains a kinase domain	Upregulated; associated with poor prognosis Promotes cancer stemness, EMT, and metastasis Enhances migration and survival Inhibition → ↓ invasion, reverses drug resistance	[4,28,29]
Kv10.1 (Eag1) (Ether-à-go-go 1, *KCNH1*)	Voltage-gated K^+^ channel (delayed-rectifier K^+^ current)	Aberrantly expressed in ~70–90% of lung cancers Promotes G_1_–S transition and proliferation Facilitates migration via coupling with Ca^2+^ channels Blockade → cell cycle arrest & reduced metastasis	[11]
KCa3.1 (IK1, *KCNN4*)	Intermediate-conductance Ca^2+^-activated K^+^ channel	Upregulated in aggressive NSCLC Promotes migration & invasion (Ca^2+^ signaling, focal adhesion turnover) Associated with EGFR-TKI resistance Inhibition → ROS generation, mitochondrial dysfunction, restored sensitivity	[2,32,33]
Nav1.5 (*SCN5A*)	Voltage-gated Na^+^ channel (tetrodotoxin-resistant sodium current)	Expressed in invasive cancer phenotypes Promotes metastasis (invadopodia formation, ECM degradation) Linked to metastatic spread (strong evidence in breast; emerging in lung) Potential cross-talk with EGFR signaling	[34,35,36]

Abbreviations: Non-small cell lung cancer (NSCLC); epithelial–mesenchymal transition (EMT); epidermal growth factor receptor (EGFR); reactive oxygen species (ROS); tyrosine kinase inhibitor (TKI). An upward arrow means higher or better, while a downward arrow means lower.

### 2.2. Potassium Channels: Modulators of Membrane Potential and Signaling

Lung cancer cells often exploit K^+^ channels to modulate their membrane potential (Vm) and indirectly control Ca^2+^ influx and cell volume. Two potassium channels stand out in lung cancer: the voltage-gated potassium channel Kv10.1 (Eag1) and the Ca^2+^-activated potassium channel KCa3.1.

#### 2.2.1. Kv10.1 (Eag1, KV10.1)

Kv10.1 is normally a neural channel (barely detectable in healthy extracerebral tissues) but is ectopically expressed in the majority of human tumors, including lung cancers. Analyses of patient samples show that ~90% of lung carcinomas have high Kv10.1 expression, whereas normal bronchial epithelium is largely negative. This oncofetal expression pattern makes Kv10.1 an attractive cancer-specific target. Functionally, Kv10.1 contributes to multiple malignant traits. It regulates the resting Vm; by conducting outward K^+^ currents, Kv10.1 can hyperpolarize the cell membrane, which paradoxically promotes cell cycling in cancer. Hyperpolarization increases the driving force for Ca^2+^ entry through channels like Orai1, thereby supporting the Ca^2+^ signals needed for cell proliferation and migration. Indeed, knockdown or pharmacologic inhibition of Kv10.1 causes G1 phase cell-cycle arrest and reduces proliferation in cancer cell lines [11,37]. For example, astemizole (a Kv10.1 blocker) or gene silencing of Kv10.1 decreased breast cancer cell proliferation and trapped cells in G1. In lung cancer, Kv10.1 is implicated in metastatic behavior: it forms a complex with Orai1 such that Kv10.1 activity is required for efficient store-operated Ca^2+^ entry driving migration. In metastatic breast cancer cells, Kv10.1 silencing reduced cell migration by ~45% and impaired invadopodial activity, a mechanism likely relevant in lung cancer metastasis as well. Clinically, high Kv10.1 expression has been correlated with more advanced disease and poorer outcomes in several cancers [11,37]. While specific data for lung cancer patient prognosis are limited, the near-universal presence of Kv10.1 in tumors suggests that it supports tumor progression. Encouragingly, Kv10.1 inhibitors have shown preclinical efficacy. Beyond astemizole, novel inhibitors (e.g., imipramine, peptide inhibitors) are in development [38,39]. Kv10.1 endows lung cancer cells with proliferative and invasive advantages by modulating electrical and Ca^2+^ signaling. Targeting Kv10.1 could selectively kill tumor cells or sensitize them to other treatments, given its low expression in normal lung tissue [11].

#### 2.2.2. KCa3.1 (IK1, KCa3.1)

KCa3.1 is an intermediate-conductance Ca^2+^-activated K^+^ channel often found in non-excitable cells (e.g., lymphocytes, epithelia). It opens in response to rises in intracellular Ca^2+^ (via calmodulin binding), allowing K^+^ efflux that hyperpolarizes the membrane. Lung cancer cells frequently upregulate KCa3.1, especially in more aggressive or therapy-resistant states [32,33]. For instance, A549 adenocarcinoma cells and other NSCLC lines express KCa3.1, with higher levels observed in invasive subclones [33]. KCa3.1 activity promotes an “invasion-competent” phenotype: by sustaining a negative membrane potential, it facilitates repeated Ca^2+^ entry spikes that drive motility and cytoskeletal dynamics. KCa3.1 has been shown to promote lamellipodial formation and migration in NSCLC cells, partly by interacting with integrin signaling pathways. Blockade of KCa3.1 (with specific inhibitor TRAM-34 or senicapoc) impairs lung cancer cell migration and can increase cell-cell adhesion via upregulating ICAM-1, thereby reducing invasive potential [2,32]. Importantly, KCa3.1 has been linked to drug resistance. A recent study by Bulk et al. (2023) found that KCa3.1 activity helps NSCLC cells resist EGFR inhibitors; inhibition of KCa3.1 resensitized resistant cells to erlotinib, an EGFR-TKI [32]. Mechanistically, blocking KCa3.1 induced a burst of mitochondrial ROS and a drop in mitochondrial membrane potential and ATP production, stressing the cancer cells metabolically. The combination of erlotinib + KCa3.1 blocker caused synergistic cancer cell killing, as the metabolic compromise from KCa3.1 inhibition exacerbated the growth signaling blockade from EGFR-TKI [2]. This positions KCa3.1 as a metabolic and survival support for lung tumors. Furthermore, epigenetic upregulation of KCa3.1 (via DNA hypomethylation) has been noted in lung cancer and correlates with worse prognosis [39], highlighting it as an epigenetically deregulated driver.

#### 2.2.3. KCa3.1 Is Also of Interest in the Tumor Microenvironment

KCa3.1 regulates functions of immune cells (such as T cells and macrophages), which can indirectly affect tumor progression [40]. In summary, KCa3.1 channels bolster lung cancer cell migration, invasion, and therapy resistance by maintaining optimal ionic conditions for these processes. They represent a viable target, and several selective inhibitors (senicapoc is in trials for other indications) could be repurposed for lung cancer. The concept of combining the KCa3.1 blockade with standard therapies (EGFR inhibitors and chemotherapy) is an attractive strategy to overcome resistance.

### 2.3. Sodium Channels: Voltage-Gated Sodium Channels and Metastatic Potential

Voltage-gated sodium channels (VGSCs) are traditionally associated with excitable tissues (nerve and muscle), but a growing body of evidence links them to cancer invasiveness. In lung cancer, VGSC expression has been documented, particularly in highly metastatic or advanced tumors [34]. One VGSC isoform, Nav1.5 (encoded by *SCN5A*), has emerged as a contributor to metastatic behavior in several cancers. While robust data on lung cancer are still emerging, by analogy to breast and colon cancer, Nav1.5 likely plays a pro-metastatic role in lung tumors. In breast cancer, *SCN5A* overexpression significantly enhanced tumor growth and lung metastasis in vivo [36]. Nav1.5 activity in cancer cells promotes the formation of invadopodia (actin-rich protrusions) and the release of proteases that degrade extracellular matrix, thereby facilitating invasion. It can also stimulate directional migration by influencing intracellular Ca^2+^ and pH (through Na^+^/H^+^ exchangers) [35]. VGSC activation leads to Na^+^ influx, which is thought to activate the Na^+^/Ca^2+^ exchanger, raising cytosolic Ca^2+^ in localized regions and triggering pro-migratory signaling cascades. In NSCLC, one study found that EGF/EGFR signaling upregulated Nav1.7 (*SCN9A*) expression and that VGSC blockade reduced invasion in EGFR-driven lung cancer cells (though this was in a cellular model). More broadly, analysis of gene expression databases indicates that certain VGSC genes (including *SCN5A*) are expressed in lung tumors and may be part of a gene signature associated with metastasis [34,41]. The presence of the Nav1.5 protein has been confirmed in some lung carcinoma samples (e.g., immunohistochemistry showing focal Nav1.5 in invasive fronts of tumors) [34]. The exact contribution of Nav1.5 to lung cancer progression is an area of active research, but by extrapolation, its activity likely aids the dynamic cytoskeletal changes and matrix degradation required for tumor cells to disseminate. Consistent with this, in various carcinomas, pharmacological inhibitors of VGSCs (like tetrodotoxin or class I antiarrhythmics) have been shown to reduce cancer cell invasion and metastasis formation [35]. It is worth noting that VGSCs might also influence tumor cell electrical coupling with the microenvironment and even affect membrane potential in ways that crosstalk with K^+^ and Ca^2+^ channel functions. Despite being a Na^+^ channel, Nav1.5 functionally interacts with K^+^ channel-driven membrane polarization, contributing to coordinated electrochemical gradients that facilitate Ca^2+^-dependent invasion. Therefore, VGSCs like Nav1.5 integrate into the broader ionic network supporting a metastatic phenotype. Given their minimal role in normal lung cells, VGSCs present a possible therapeutic target for metastasis suppression.

**Figure 2:**
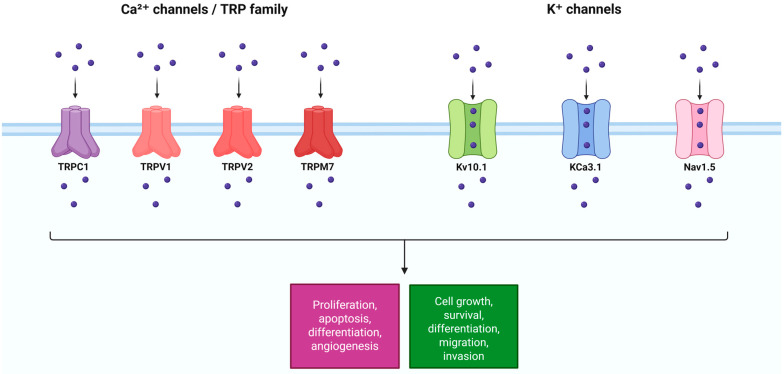
Ion channels as active central oncogenic drivers in lung cancer cells. Calcium, potassium, and sodium channels regulate intracellular Ca^2+^ oscillations, membrane polarization, and invasive behavior. Their dysregulation directly contributes to lung cancer progression, supporting the concept that ion channels function as central oncogenic drivers rather than passive conduits. Although Nav1.5 is a voltage-gated sodium channel, it is positioned alongside potassium channels in this schematic to reflect its functional coupling with K^+^ channel-mediated membrane potential regulation, which collectively governs Ca^2+^ influx and invasive behavior in lung cancer cells. This figure was created via Biorender Premium License by Fahrul Nurkolis.

Lung cancer cells repurpose a variety of ion channels to meet the demands of unchecked growth and metastasis. TRP channels provide calcium signals for proliferation and survival; K^+^ channels fine-tune membrane voltage and calcium entry to promote cell cycle and motility; Na^+^ channels contribute to invasive capacity (Figure 2). Crucially, these channels do not act in isolation; they intersect with classical oncogenic pathways (EGFR, PI3K/AKT, etc.) and with GPCR signaling.

## 3. GPCR Signaling Pathways in Lung Cancer

**Figure 3:**
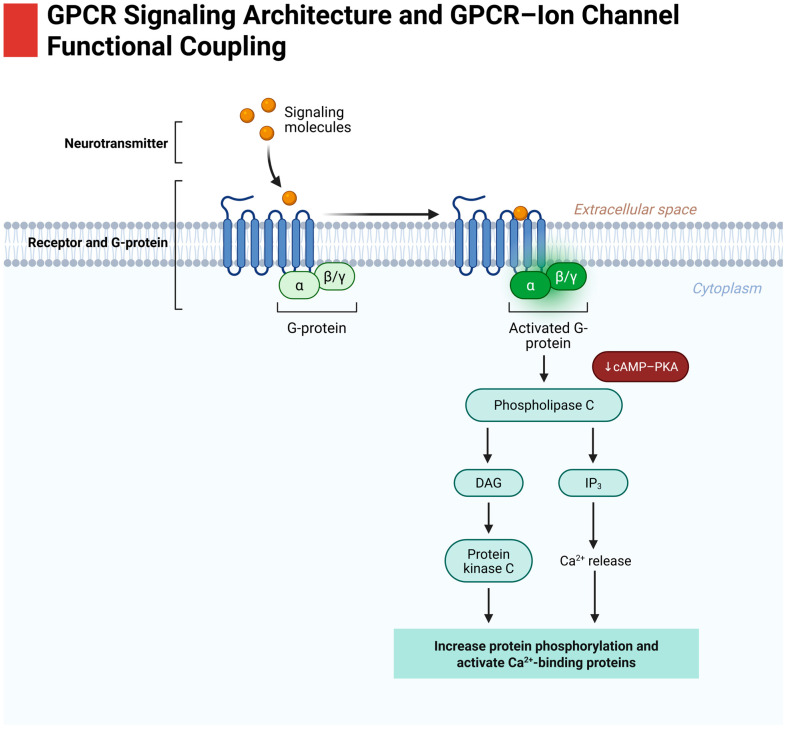
GPCR signaling architecture and GPCR-ion channel functional coupling in lung cancer. GPCR activation orchestrates multiple signaling cascades through classical G protein pathways and β-arrestin scaffolding. Gβγ subunits and membrane microdomains enable direct coupling between GPCRs and ion channels, generating localized Ca^2+^ microdomains that regulate oncogenic signaling outputs. This figure was created via Biorender Premium License by Fahrul Nurkolis.

GPCRs comprise the largest family of cell-surface receptors and are key transducers of extracellular cues (chemokines, neurotransmitters, and metabolites) into intracellular signals [42]. In cancer, GPCRs and their ligands in the tumor microenvironment can hijack normal signaling to favor tumor survival, angiogenesis, and metastasis (Figure 3). Lung cancers often exhibit dysregulation of multiple GPCR-mediated pathways: Autocrine and paracrine signals (e.g., prostaglandins, LPA, S1P, chemokines) activate GPCRs on tumor cells, stimulating downstream oncogenic cascades [43,44,45]. This outlines the major GPCR signaling modules relevant to lung cancer, namely the canonical Gαs/cAMP/PKA pathway, Gαq/PLCβ/Ca^2+^ pathway, and β-arrestin-mediated signaling, and then focuses on specific GPCRs implicated in lung cancer progression, including the classical cannabinoid receptors (CB1 and CB2), the “non-classical” cannabinoid-related receptor GPR55, adenosine receptors, and the chemokine receptor CXCR4.

### 3.1. Canonical GPCR Signaling Pathways (Gα and β-Arrestin)

Upon ligand binding, GPCRs undergo conformational changes that enable coupling to heterotrimeric G proteins. The Gα subunit (with bound GTP) and Gβγ dimer dissociate and modulate various effectors.

#### 3.1.1. Gαs cAMP/PKA Pathway

Gs-coupled GPCRs (e.g., β-adrenergic receptors, adenosine A2A receptor) activate adenylate cyclase, raising intracellular cAMP. cAMP, in turn, activates protein kinase A (PKA) and EPAC (exchange protein activated by cAMP), which phosphorylate targets controlling metabolism, gene transcription, and cell proliferation. In lung cancer, the cAMP/PKA pathway can have context-dependent effects. Moderate cAMP signaling is often pro-survival (PKA can phosphorylate CREB, promoting expression of pro-proliferative genes), and some tumors upregulate Gs-coupled GPCRs or adenylate cyclase isoforms. However, excessive cAMP can induce growth arrest or differentiation in certain contexts. For example, prostaglandin E2 signals through Gs-coupled EP2/EP4 receptors in lung tumors, elevating cAMP and promoting angiogenesis and tumor growth (via PKA-mediated VEGF expression). On the other hand, stable activation of PKA can inhibit the cell cycle by stabilizing p27^Kip1^ or by antagonizing MAPK signaling. Thus, Gs signals can both support tumor growth and, if hyperactivated, impose constraints. The net outcome depends on the balance of downstream effectors. Many lung cancers produce autocrine adenosine, which acts on A2B (Gs) receptors to stimulate cAMP and drive EMT and metastasis [46,47]. Therapeutically, modulation of cAMP is complex. PDE4 inhibitors, for instance, raise cAMP and have been tested as anti-inflammatories in lung cancer patients, with unclear anti-tumor benefits.

#### 3.1.2. Gαi/o Inhibition of cAMP and PI3K Activation

Gi-coupled GPCRs (e.g., CB1, CB2, somatostatin receptors, adenosine A3) inhibit adenylate cyclase, thus lowering cAMP/PKA activity. This can relieve PKA-mediated inhibition of certain pathways (PKA often restrains the Raf/MEK/ERK cascade, so less PKA can allow stronger ERK activation). Gi also frees βγ subunits that directly activate phosphoinositide-3-kinase (PI3K) and PLCβ, or open ion channels (discussed later). In lung cancer, many overexpressed GPCRs (like CXCR4, prostaglandin E receptors EP3, and certain dopamine receptors) signal through Gi. Activation of Gi can favor tumor cell survival by unleashing the PI3K-AKT pathway: Gβγ subunits from Gi recruit PI3Kγ to the membrane, leading to PIP3 production and AKT activation. Indeed, some studies show that Gi-biased GPCR activation (for instance, by cannabinoids at CB2) results in AKT and ERK pathway stimulation in cancer cells [7]. However, prolonged Gi signaling can also be growth-inhibitory via cAMP reduction; for example, activating CB1/CB2 in lung cancer was found to decrease phosphorylated AKT and ERK over time, contributing to apoptosis [7,48]. Thus, the kinetics and context of Gi signaling determine outcomes. Overall, Gi/o pathways in tumors often promote chemotaxis and metastasis (through PI3Kγ/Rac activation) and can protect cells from apoptosis initially. Many chemokine receptors (CXCR2 and CXCR4) are Gi and are known to drive lung cancer cell migration and invasion by this mechanism [49,50].

#### 3.1.3. Gαq/11 PLCβ/Ca^2+^/PKC Pathway

Gq-coupled GPCRs (e.g., GPCRs for thrombin, acetylcholine M3, LPA1/3, and GPR55 in some contexts) activate phospholipase C-β (PLCβ). PLCβ hydrolyzes PIP2 into IP3 and DAG. IP3 triggers Ca^2+^ release from the endoplasmic reticulum, raising cytosolic Ca^2+^, while DAG (plus Ca^2+^) activates protein kinase C (PKC) isoforms. The Gq → PLCβ → PKC pathway is frequently pro-oncogenic. In lung epithelial cells, chronic Gq signaling (e.g., by lysophosphatidic acid or endothelin) induces proliferation via PKC and the ERK pathway and can stimulate matrix metalloproteinase secretion, aiding invasion. Ca^2+^ released by IP3 can activate Ca^2+^-dependent transcription factors (NFAT, calmodulin-CAMK pathways) that promote cell cycle progression and angiogenesis factor production. For example, bombesin (a neuropeptide) acts on Gq-coupled GRP receptors in SCLC, causing a burst of Ca^2+^ and PKC activation that drives mitogenesis. Gq signaling also intersects with EGFR: Gq-coupled GPCRs can cause “EGFR transactivation” by ADAM metalloprotease-dependent shedding of EGF ligands [7]. In lung cancer, this mechanism is seen with GPCR agonists like bradykinin and LPA, which, via Gq, cause release of HB-EGF and consequent EGFR activation. GPR55, an oncogenic GPCR overexpressed in some cancers, primarily signals through Gq and G12/13, raising intracellular Ca^2+^ and activating Rho GTPases; this has been linked to enhanced cancer cell motility and metastasis [51]. In short, Gq pathways potentiate lung tumor growth and invasive capabilities by mobilizing Ca^2+^ and PKC-driven oncogenic programs. Inhibiting Gq-linked GPCRs or downstream PLC/PKC has shown anti-tumor effects in preclinical models (e.g., PLCβ inhibitors reduce NSCLC growth in some studies).

#### 3.1.4. β-Arrestin Scaffold Signaling

In addition to G proteins, activated GPCRs recruit β-arrestins (β-arr1 and β-arr2), which classically mediate receptor desensitization and internalization. However, β-arrestins also function as scaffold proteins initiating G protein-independent signaling. β-arrestin-bound GPCRs can form signalosomes that activate ERK1/2, p38 MAPK, AKT, and others in distinct temporal/spatial patterns. This is termed biased agonism when a ligand preferentially signals via β-arrestin vs. G proteins. In cancer, β-arrestin pathways can have unique outcomes. β-arrestin-bound ERK may localize to the cytosol/nucleus differently than G protein-activated ERK, affecting cell fate decisions. Some GPCRs (e.g., CXCR4, AngII receptor AT1), when stimulated by certain ligands, use β-arrestin to drive sustained ERK or NF-κB signaling that promotes migration or survival. In lung cancer, β-arrestin-1 has been shown to associate with CXCR4 and facilitate its pro-migratory signaling, and knocking down β-arrestin-1 reduced CXCR4-driven invasion [52]. Another example is as follows: The β2-adrenergic receptor (β2AR) can form complexes with CXCR4, and agonist-induced β-arrestin recruitment to β2AR/CXCR4 heteromers was found to inhibit Gα13/RhoA signaling from CXCR4, thereby reducing chemotaxis [52,53]. This indicates β-arrestin can either propagate signals or act as a cross-talk modulator depending on the context. Regarding cannabinoid receptors, CB1 and CB2 are known to signal via β-arrestins and Gi/o. β-arrestin-biased activation of CB1 has been implicated in cellular outcomes distinct from G protein pathways (e.g., possibly contributing to anti-apoptotic effects in neurons, but in cancer, the role is still being elucidated). What is clear is that the balance of G vs. β-arrestin signaling from GPCRs can influence cell proliferation and apoptosis. Some studies suggest that β-arrestin signaling might favor pro-apoptotic pathways in cancer: For instance, cannabinoid analogues that strongly recruit β-arrestin were shown to induce more ER stress and apoptosis in certain cancer cells [7]. On the other hand, β-arrestin scaffolds can activate AKT and inhibit apoptosis (β-arrestin-1 can scaffold PI3K in some contexts). Thus, β-arrestin effects in lung cancer are complex and receptor-specific. Nonetheless, the concept of biased GPCR agonism, designing drugs to preferentially activate either G protein or β-arrestin routes, holds promise for tuning desired anti-cancer responses while minimizing side effects.

GPCR signaling in lung cancer is multi-dimensional. Gs/PKA, Gi/PI3K, Gq/PLC, and β-arrestin signals intersect with each other and with receptor tyrosine kinase pathways to shape tumor cell behavior. Next, we focus on specific GPCRs of high relevance to lung cancer, particularly those in the endocannabinoid system or influenced by cannabinoids.

### 3.2. Key GPCRs in Lung Cancer and Their Roles

#### 3.2.1. Cannabinoid Receptor 1 (CB1) and Cannabinoid Receptor 2 (CB2)

These are the classical receptors for endocannabinoids and phytocannabinoids. CB1 (encoded by *CNR1*) is predominantly Gi/o-coupled and highly expressed in the central nervous system, while CB2 (*CNR2*) is also Gi-coupled and enriched in immune tissues. In lung cancer, both CB1 and CB2 are present on tumor cells. A study of NSCLC patient tumors found high CB1 mRNA/protein expression in ~24% of cases and CB2 in ~55% [7]. Interestingly, patients with high CB1 (and/or high CB2) tumor expression had improved survival compared to low expressors, suggesting that an active endocannabinoid system might restrain tumor progression [9]. This aligns with preclinical data: Activation of CB1/CB2 receptors has generally anti-proliferative and pro-apoptotic effects in lung cancer models [9,48]. For example, Δ^9^-THC (a mixed CB1/CB2 agonist) and CBD (a CB1/CB2 modulator) both inhibited NSCLC cell growth in vitro, with a combination of THC + CBD synergistically reducing proliferation and migration [9]. Mechanistically, CB1/CB2 activation in NSCLC cells leads to Gi signaling: decreased cAMP, which can cause cell cycle arrest, and βγ-mediated signaling that can trigger ERK and p38 MAPK-mediated apoptosis. THC and synthetic agonists have been shown to elevate ceramide (through stimulation of sphingomyelin breakdown or de novo synthesis), which initiates an ER stress-related pro-death pathway in cancer cells. In lung cancer specifically, cannabinoids induced apoptosis via both caspase activation and autophagy, accompanied by inhibition of PI3K-AKT-mTOR signaling [7]. Notably, CB1/CB2 engagement can downregulate key survival genes in NSCLC. Milian et al. (2020) [9] demonstrated that THC or CBD treatment of NSCLC cells not only slowed proliferation but also decreased EGFR expression at the mRNA level. The THC/CBD combination restored an epithelial phenotype, increasing E-cadherin and reducing vimentin, effectively reversing EMT [9]. This indicates CB1/2 activation intersects with growth factor signaling and EMT regulation, both central to lung tumor progression. On the immunological side, CB2 activation on tumor-associated immune cells might modulate anti-tumor immunity (CB2 on myeloid cells generally dampens inflammatory responses). However, in the context of cancer, CB2 agonism has shown beneficial effects, like reducing pro-tumor macrophage activity and inhibiting angiogenesis through CXCR4 cross-talk. Importantly, CB1 vs. CB2 may have divergent roles: CB1 activation (especially in the CNS) has psychoactive side effects that limit direct use of CB1 agonists systemically, whereas CB2 is non-psychoactive and primarily peripheral. Many lung cancers express both receptors, but some data suggest that CB2 is upregulated in more advanced malignancies, which is possibly a cellular attempt to counteract tumor growth (as CB2 agonists tend to be growth-inhibitory). In any case, both receptors are promising targets: Agonists or positive modulators of CB1/2 could be therapeutic, and indeed, a CB2-selective agonist (JWH-133) significantly reduced lung tumor growth and metastasis in murine models, reportedly by inducing cancer cell apoptosis and impeding angiogenesis [7,54,55]. Overall, CB1 and CB2 act as tumor-suppressive GPCRs when activated, and lung tumors may downregulate their endogenous activity as they progress. Restoring or enhancing CB1/2 signaling (via exogenous cannabinoids) is a key rationale for cannabinoid-based lung cancer therapies.

#### 3.2.2. GPR55

GPR55 is an orphan GPCR often regarded as an “unconventional cannabinoid receptor” because some cannabinoids (e.g., certain CBD analogs) can bind it, though its endogenous ligand is likely lysophosphatidylinositol (LPI). GPR55 couples to G12/13 and Gq, activating RhoA and PLC pathways. It has emerged as a pro-tumorigenic receptor in multiple cancers [51]. In lung cancer, GPR55 is less studied than in breast or pancreatic cancer, but evidence suggests that it plays a role in tumor progression. MicroRNA-675-5p was found to target GPR55; in lung cancer cells, downregulation of miR-675-5p led to increased GPR55 expression and was associated with enhanced proliferation and metastasis. Conversely, restoring miR-675-5p suppressed lung cancer progression by reducing GPR55 levels [56]. This implies that GPR55 drives malignant phenotypes when upregulated. GPR55 signaling can stimulate cancer cell migration and angiogenesis via downstream effectors like ROCK (from RhoA activation) and Ca^2+^ mobilization. In other models, GPR55 overexpression correlates with higher tumor aggressiveness, and GPR55 knockout or inhibition curtails tumor growth [51]. Notably, cannabinoids can modulate GPR55: CBD is an antagonist/inverse agonist at GPR55, and THC is reported to be a partial agonist. The presence of GPR55 in lung tumors offers another axis by which cannabinoids may act. By blocking GPR55 (as CBD does), one could inhibit LPI-induced protumor signals. No direct clinical data yet link GPR55 expression with lung cancer patient outcomes, but pan-cancer analyses list *GPR55* among GPCR genes upregulated in certain tumors. Akin to CB receptors, GPR55 might interact with other receptors; for example, it has been suggested that CB2 and GPR55 form heteromeric complexes in some cells, and CBD can bias signaling through such heteromers [57]. In immune contexts, GPR55 deficiency was shown to affect neutrophil function in lung injury models, which could indirectly influence tumor inflammation [58]. Overall, GPR55 appears to function as a pro-oncogenic GPCR in lung cancer. Antagonizing it (e.g., with CBD or specific inhibitors) may tilt the balance toward tumor suppression. Given its coupling to Ca^2+^ pathways, GPR55 also exemplifies GPCR-ion channel cross-talk: its activation elevates Ca^2+^, which can modulate TRP channels and other Ca^2+^-dependent processes in cancer cells.

#### 3.2.3. A2A, A2B, A1, and A3

Tumors, including lung cancers, often accumulate high levels of adenosine in their microenvironment (due to hypoxia-induced ATP release and ectonucleotidase activity). Adenosine is a potent immunosuppressive agent and signals through four GPCR subtypes: A1 and A3 (Gi-coupled) and A2A and A2B (Gs-coupled). In the context of lung cancer, adenosine receptors are important at the interface of tumor and immune cells. A2A receptors on T cells and NK cells strongly suppress anti-tumor immunity when activated by adenosine (cAMP/PKA in immune cells leads to reduced cytokine release and cytotoxicity). Many lung tumors upregulate CD39/CD73 ectonucleotidases, generating adenosine that acts on A2A to protect the tumor from immune attack [59]. Blockade of A2A receptors (e.g., with small molecules or caffeine analogs) is being explored to enhance the efficacy of immunotherapy in lung cancer patients. On the tumor cells themselves, A2B receptors are often expressed and can promote tumor progression by dual mechanisms: Gs/PKA signaling in cancer cells can increase VEGF and IL-8 production (stimulating angiogenesis and growth), and A2B on immune cells skews toward a pro-tumoral, macrophage M2 phenotype. In NSCLC, high A2B expression was linked to increased metastasis and poor prognosis in some studies due to its role in driving EMT and invasion via cAMP→EPAC→RAP1 signaling. A3 receptors are somewhat different; A3 is Gi-coupled, and in some tumor models (including lung), selective A3 agonists have shown anti-cancer effects. An A3 agonist (CF102, namodenoson) induced apoptosis in NSCLC cell lines by inhibiting the PI3K-AKT pathway and downregulating cyclin D, possibly through Gi-mediated effects [60]. A3 activation can also inhibit angiogenesis by decreasing VEGF. Thus, A3 is considered a tumor-suppressor GPCR in contrast to A2A/A2B. Notably, cannabinoids can influence adenosine signaling. CBD and THC have been reported to increase extracellular adenosine levels by inhibiting its cellular uptake (via ENT transporters) [61]. This paradoxically might bolster A2A/A2B signaling. In a non-cancer context, CBD’s anti-inflammatory effects are partly attributed to elevated adenosine activating A2A on immune cells. In cancer, however, increased adenosine could be detrimental by suppressing the immune response. This suggests that while cannabinoids have direct tumor-killing properties, their net effect on the immune-tumor interplay might be complex if they raise adenosine. One strategy would be to use cannabinoids in combination with A2A antagonists to ensure that the immune system remains active. Alternatively, designing cannabinoid analogs that do not impact adenosine transport could avoid this issue. In summary, adenosine receptors in lung cancer present a double-edged sword: A2A/A2B largely facilitate tumor immune evasion and progression, whereas A3 (and possibly A1) can oppose tumor growth. Any cannabinoid-based therapy in lung cancer should consider adenosine pathway interactions to maximize anti-tumor efficacy.

#### 3.2.4. CXCR4 (CXC Chemokine Receptor 4)

CXCR4, a Gi-coupled chemokine receptor for CXCL12 (SDF-1), is a critical mediator of metastasis in many cancers. In lung cancer, CXCR4 is frequently overexpressed and guides tumor cells to metastatic niches (like bone marrow, brain) where CXCL12 is abundant [49,50]. High CXCR4 expression in NSCLC is associated with increased invasion, lymph node spread, and worse survival [50]. CXCL12/CXCR4 signaling activates MAPK and PI3K/AKT pathways via Gi and β-arrestin, enhancing tumor cell migration, EMT, and survival under stress. Moreover, CXCR4 in tumor cells promotes angiogenesis and recruits immunosuppressive cells to the tumor microenvironment. Because of its pivotal role, CXCR4 has been a therapeutic target for small molecule antagonists (AMD3100), and peptide inhibitors are known to reduce metastasis in models and are in clinical trials. A fascinating aspect is the crosstalk between CXCR4 and cannabinoid signaling. Studies have found that CB2 receptors can heterodimerize with CXCR4, forming CXCR4-CB2 complexes on cell membranes [62,63]. When both receptors are co-activated (e.g., by CXCL12 and a CB2 agonist), the heterodimer preferentially signals through CB2′s Gi in a way that antagonizes the typical CXCR4 response [52,62]. Specifically, simultaneous stimulation led to reduced Gα13/RhoA activation and diminished migration in cancer cells, as the CB2 βγ and β-arrestin recruitment interfered with CXCR4′s signaling [7,52]. In practical terms, activating CB2 can inhibit CXCR4-driven metastasis. For example, activation of CB2 by cannabinoids was shown to downregulate CXCR4 expression on cancer cells and impede their chemotaxis toward CXCL12. Gelfand et al. (2025) reported that a combination of CBD with cannabidivarin (CBDV) significantly reduced CXCR4 surface expression on immune cells via CB2 activation, leading to impaired chemotaxis; this effect was absent in CB2-knockout conditions [64]. By analogy, in lung cancer cells, CB2 activation likely triggers internalization or degradation of CXCR4, or functionally sequesters it in heterodimers, thereby reducing metastasis. Additionally, CB2-CXCR4 interaction can affect angiogenesis: One study noted that CB2 activation can inhibit tumor angiogenesis by binding to CXCR4 and inducing the release of TIMP-1 (a metalloproteinase inhibitor) [7]. Therefore, CXCR4 is a prime example of how a pro-metastatic GPCR can be modulated by cannabinoid receptor signaling. Combining CXCR4 antagonists with cannabinoids or using CB2 agonists might achieve a synergistic anti-metastatic effect. CXCR4 is a master regulator of lung cancer metastasis, and its functional suppression via CB2-cannabinoid pathways highlights a promising therapeutic avenue.

#### 3.2.5. Other GPCRs

While the above are most pertinent to cannabinoid interactions, lung cancer is also influenced by many other GPCRs: the protease-activated receptor PAR1 (thrombin receptor), which drives invasion via Gq; the formyl peptide receptor (FPR1), which can promote chemotaxis of cancer cells; and the prostanoid receptors (EP1-EP4 for PGE2), where EP4 (Gs) and EP1 (Gq) have been implicated in NSCLC progression. Notably, EP2 and EP4 (Gs-coupled) in tumor cells contribute to immunosuppression and were shown to induce resistance to immune checkpoint therapy [43]. A comprehensive review is beyond our study’s scope, but it is clear that GPCR signaling forms a network that intertwines with ion channel signaling to regulate lung cancer cell fate. We next examine how these GPCRs physically and functionally couple to ion channels in specialized microdomains.

## 4. GPCR-Ion Channel Coupling in Lung Cancer: Gβγ, Microdomains, and Signal Integration

GPCRs and ion channels do not operate in isolation within the cell membrane; they often co-localize and interact in discrete membrane microdomains (such as lipid rafts and caveolae) and share signaling components, allowing for tightly regulated cross-talk. In lung cancer cells, these interactions enable rapid and spatially confined signaling events. Several mechanisms facilitate GPCR-channel coupling:

### 4.1. G-Protein βγ Subunits Direct Channel Modulation

Upon GPCR activation, freed Gβγ dimers can directly bind and modulate ion channels. This is well-established in excitable cells (e.g., Gβγ from Gi binds to and inhibits voltage-gated Ca^2+^ channels, or opens GIRK K^+^ channels). In cancer cells, similar principles apply. For instance, Gβγ subunits released from GPCRs can activate PLCβ (independently of Gαq), leading to IP3 production and Ca^2+^ release that activates store-operated Ca^2+^ channels like TRPC1 [22]. Gβγ can also directly gate certain TRP channels: Studies have shown Gβγ binding sites on TRPC channels, modulating their open probability. In lung cancer, a chemokine like CXCL12, which binds to CXCR4 (Gi), leads to Gβγ activation of PI3K and PLCβ, which in turn can trigger Ca^2+^ influx through TRPC channels, integrating chemokine receptor signaling with Ca^2+^ oscillations that promote migration [65,66]. Gβγ also interacts with some K^+^ channels; for example, Gβγ can bind and open Kir3 (GIRK) channels. While GIRKs are mostly neuronal, some are expressed in small-cell lung cancer. If present, Gβγ from somatostatin or opioid receptors could hyperpolarize the cell via GIRKs, potentially affecting neuroendocrine tumor cell excitability [67]. Moreover, Gβγ-PI3K signaling can indirectly modulate ion channels by PIP3 generation. PIP3 can bind to and open certain channels or recruit scaffolds that affect channel trafficking.

### 4.2. Lipid Rafts and Caveolae as Signaling Microdomains

Lipid rafts are cholesterol- and sphingolipid-rich membrane microdomains that concentrate certain proteins, creating a platform for efficient signaling. Many GPCRs and ion channels partition into rafts. Caveolin-1, a major component of caveolae (flask-shaped rafts), is highly expressed in some lung cancers and acts as a scaffold. CB1 cannabinoid receptors, for example, are known to localize in lipid rafts [68,69]. In a classic study, disrupting rafts with methyl-β-cyclodextrin (MCD) increased CB1 receptor mobility and G-protein coupling, enhancing CB1 signaling (GTPγS binding and downstream AC/MAPK activity) [70]. This suggests that rafts normally sequester CB1 and limit its signaling range. At the same time, TRPV1 and endocannabinoid enzymes (like FAAH) did not change localization with MCD, indicating selective raft association [70]. In lung cancer cells, rafts likely cluster GPCRs (like CB1/CB2, CXCR4, EGFR) together with ion channels (like TRPCs, Orai1, KV10.1) to form signalosomes. For instance, caveolin-1 can bind KV10.1 and stabilize it in membrane microdomains, where it colocalizes with integrins and growth factor receptors during cell migration [11]. Disruption of caveolae was shown to impair KV10.1-mediated enhancement of lung cancer cell invasiveness in some studies, underlining the importance of microdomain context. Lipid rafts also host Na^+^/Ca^2+^ exchangers, pumps, and scaffolding kinases, meaning a high local concentration of signaling molecules. The interplay between CB receptors and rafts is particularly noteworthy: one report suggested that anandamide-induced apoptosis in glioma cells required CB1 to be outside of rafts when rafts were disrupted, CB1 signaling increased, but paradoxically, apoptosis decreased because CB1′s prosurvival signaling (via MAPK) became too strong [7,70]. This illustrates that raft localization can qualitatively alter signal output. In lung cancer, we might find that CB2 in rafts vs. outside rafts could determine whether it preferentially engages CXCR4 heterodimers or not. Indeed, certain CB2 agonists could be designed to shift receptor distribution between microdomains, biasing the interaction with specific channels or partners.

### 4.3. GPCR-Ion Channel Complexes and β-Arrestin

Some GPCRs physically assemble with ion channels in macromolecular complexes, often via scaffolding proteins. For example, β2-adrenergic receptors form complexes with L-type Ca^2+^ channels in cardiomyocytes through A-kinase anchoring proteins (AKAPs) that tether PKA near the channel for rapid modulation. In lung cancer, AKAPs (like AKAP79/150) could tether PKA and calcineurin to Ca^2+^ channels (CRAC channels) near β2AR or other GPCRs, creating a local feedback loop [71,72]. Another example is the formation of GPCR heterodimers that include an ionotropic component. There is evidence that CB1 can heterodimerize with the dopamine D2 receptor, modulating Ca^2+^ channel regulation in neurons; similarly, CB2-CXCR4 heterodimers in cancer cells could influence the Ca^2+^ fluxes needed for chemotaxis. Also, TRPV1 has been found to interact with the μ-opioid receptor in neurons; a comparable interplay in cancer could exist between TRP channels and GPCRs [73,74].

In addition to the pathways described above, β-arrestins can scaffold not only kinases but also ion channels or channel regulators. β-arrestin-2 has been shown to bind and regulate TRPV4 channel trafficking in some cell types. In SCLC, which often expresses β-arrestin and various TRP channels, one could envision β-arrestin linking an activated GPCR to a TRP channel, perhaps ensuring that the channel is closed after an initial Ca^2+^ burst (desensitization of TRP via β-arrestin recruitment is a possibility). Additionally, β-arrestin could recruit phosphodiesterases to the vicinity of cAMP-sensitive ion channels (like HCN channels, if any in cancer cells), thus integrating GPCR as a second messenger and improving ion channel control [75].

### 4.4. Caveolin and Channel Regulation

Caveolin-1 (Cav1) often binds to motifs in GPCRs and channels, modulating their function. In lung cancer, Cav1 is something of a double-edged sword in some NSCLC contexts; high Cav1 correlates with worse outcomes (as it fosters EMT and metastasis), while in other contexts, Cav1 acts as a tumor suppressor (reducing growth factor signaling). Regardless, Cav1′s presence means that many GPCRs and channels reside in caveolae [76,77,78]. For example, store-operated Ca^2+^ entry components (STIM1 and Orai1) cluster at ER-plasma membrane junctions that are often enriched in caveolae. STIM1 itself can bind TRPC1 and scaffolds, and caveolin may stabilize this assembly. A scenario can be drawn where a Gq GPCR (say P2Y2 nucleotide receptor) in caveolae triggers IP3-mediated Ca^2+^ store release; STIM1 senses this and opens TRPC1/Orai1; meanwhile, Cav1-bound β-arrestin or kinases coordinate feedback. If we introduce a cannabinoid receptor into this mix, say CB2, which might also localize in lipid rafts, activation of CB2 could perturb the microdomain localization or the activity of the complex. Indeed, there is evidence that CBD can disrupt lipid raft architecture at high concentrations, potentially affecting the signaling islands where GPCRs and channels interact [2,79].

Lung cancer cells utilize microdomain-localized complexes to ensure efficient GPCR-ion channel crosstalk. Gβγ subunits from GPCRs directly regulate channels or indirectly via PLCβ/PI3K; lipid rafts concentrate receptors and channels for precise signaling; and scaffolds like caveolin-1 and β-arrestin organize these interactions. Understanding it is crucial because cannabinoids can perturb or exploit these couplings, for instance, by causing CB2-CXCR4 heterodimers (antagonizing CXCR4′s normal signaling) or by altering lipid raft composition (thereby changing channel activity).

## 5. Cannabinoids as Modulators of Ion Channels and Biased GPCR Ligands

**Figure 4:**
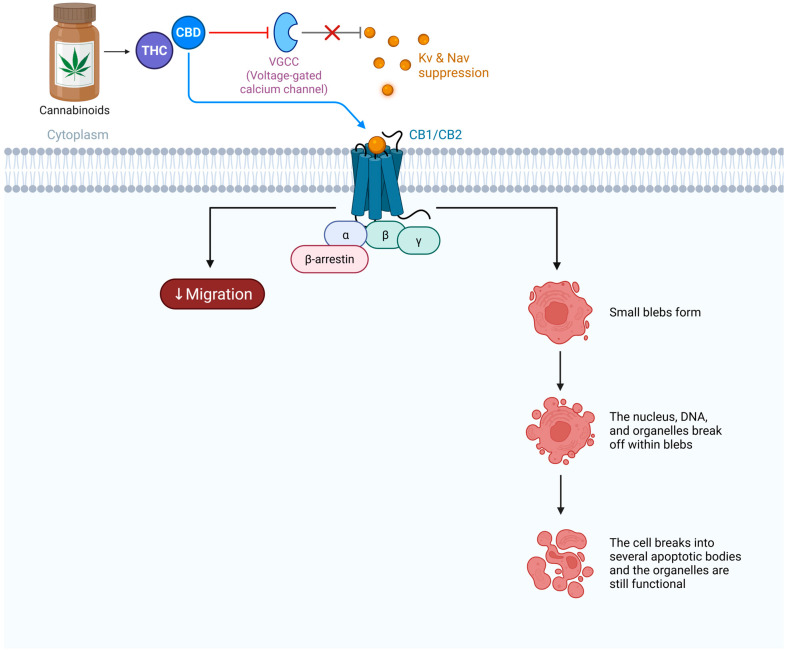
Cannabinoids as electrophysiological modulators of ion channels and GPCR signaling. Cannabinoids modulate both GPCR activity and ion channel function, reshaping ionic fluxes and downstream signaling. Through biased CB1/CB2 signaling and direct TRP channel activation, cannabinoids induce Ca^2+^ dysregulation, cell cycle arrest, and suppression of invasive phenotypes. This figure was created via Biorender Premium License by Fahrul Nurkolis.

Cannabinoids are pleiotropic compounds capable of influencing multiple signaling targets. In lung cancer cells, cannabinoids engage not only their canonical GPCRs (CB1/CB2) but can also interact with various ion channels and even nuclear receptors (e.g., PPARs). This multimodal activity positions cannabinoids as electrophysiological modulators; they can induce changes in cellular excitability, calcium homeostasis, and second messenger levels in ways that conventional single-target drugs cannot [80,81,82]. Figure 4 and this section discuss how cannabinoids modulate ion channel function and how they can act as biased ligands at GPCRs, meaning that they preferentially activate certain signaling pathways over others. These properties are key to the “rewiring” of signaling that occurs when lung cancer cells are exposed to cannabinoids.

### 5.1. Cannabinoids and Ion Channel Modulation

Several ion channels implicated in lung cancer are directly sensitive to cannabinoids or endocannabinoid metabolites:

#### 5.1.1. Cannabinoid Receptors and Classical GPCR Signaling

Many cannabinoids (endogenous and phytocannabinoids) can activate or inhibit TRP family ion channels. A prime example is TRPV1. The endocannabinoid anandamide (AEA) is a partial agonist at TRPV1; at micromolar concentrations, AEA binds to the capsaicin site on TRPV1, causing channel opening and Ca^2+^ influx. In sensory neurons, this contributes to pain modulation, but in cancer cells, AEA-induced TRPV1 activation can trigger Ca^2+^-dependent apoptosis. Indeed, one mechanism by which AEA or THC kills tumor cells is through TRPV1-mediated Ca^2+^ overload and subsequent mitochondrial dysfunction [7,83]. Capsaicin-like cannabinoids such as arvanil (a synthetic hybrid of AEA and vanilloid moiety) have been shown to induce lung cancer cell death via TRPV1 activation and downstream calpain/caspase pathways [16]. CBD can also activate TRPV1, albeit less potently than TRPV2. Activation of TRPV1 by cannabinoids initially raises Ca^2+^ and can lead to excitotoxicity, but prolonged activation often causes TRPV1 desensitization (via Ca^2+^-dependent calmodulin and phosphatase actions). This desensitization might paradoxically be beneficial in chronic settings by silencing TRPV1′s pro-tumor signals (given TRPV1′s role in NSCLC growth via IGF1R, as discussed). So cannabinoids might exert a biphasic effect on TRPV1: acute activation (Ca^2+^ spike) leading to apoptosis in some cells, followed by desensitization/downregulation of TRPV1, which could reduce its tumor-promoting activity. Consistent with this, treating lung cancer cells with vanilloids or high-dose CBD causes an early Ca^2+^ transient (pro-apoptotic) and later diminishes TRPV1 expression [3,84,85].

CBD is one of the most potent activators of TRPV2 identified (EC50 in the low micromolar range). In cisplatin-resistant lung cancer cells, CBD opens TRPV2 channels, allowing a sustained Ca^2+^ influx that initiates cell death cascades. Importantly, this action can restore chemosensitivity: by killing off the subpopulation of cells that had become drug-resistant (often cancer stem-like cells), CBD via TRPV2 essentially “re-sensitizes” the tumor to chemotherapy. Other phytocannabinoids like Δ^9^-THC and CBG (cannabigerol) may also modulate TRPV2, though CBD is best documented. Additionally, some synthetic cannabinoids (e.g., WIN55, 212-2) have been shown to indirectly enhance Ca^2+^ influx in certain cancer cells, possibly via TRPV2 activation or by upregulating its expression (there is evidence that some cannabinoid treatments increase TRPV2 levels in glioma cells, facilitating chemotherapeutic uptake). In lung cancer, it was observed that TRPV2 expression itself is beneficial for prognosis; cannabinoids may exploit this by specifically targeting TRPV2-high cells for destruction [10,86,87].

There are other TRP channels that cannabinoids affect. TRPM8 (a cold-activated Ca^2+^ channel) is inhibited by THC and CBD. If lung cancer cells express TRPM8 (prostate and lung are among those that can), THC, acting as a TRPM8 antagonist, might reduce any pro-survival Ca^2+^ signals from TRPM8. TRPA1, another Ca^2+^ permeable channel, can be activated by CBD. While TRPA1 is more relevant in neurogenic inflammation, interestingly, it is overexpressed in SCLC, where it prevents apoptosis [88]. Agonism of TRPA1 by cannabinoids like CBD could overwhelm SCLC cells with Ca^2+^, promoting cell death, which is a hypothesis that remains to be fully tested.

#### 5.1.2. Non-Classical GPCR Targets in Cannabinoid Signaling

In excitable cells, CB1 receptor activation leads to Gi βγ-mediated inhibition of N-type and P/Q-type Ca^2+^ channels, reducing neurotransmitter release. In lung cancer (non-excitable, generally no action potentials), VGCCs are less prominent, but some NSCLC cells express T-type Ca^2+^ channels or L-type channels (especially neuroendocrine variants). If present, CB1 activation by THC could inhibit these channels via Gβγ, potentially reducing Ca^2+^-dependent proliferation signals. Conversely, cannabinoids might indirectly reduce the expression of certain Ca^2+^ channels. There is evidence that in pancreatic cancer cells, cannabinoid treatment downregulated the Cav3.2 T-type channel, contributing to cell cycle arrest (since T-type channels help drive the G1-S transition by providing Ca^2+^ for cyclin E/CDK2 activation). Similar effects could occur in lung cancer cells that utilize T-type currents for proliferation. Cannabinoids could suppress those currents and thus slow the cell cycle [89,90,91].

Cannabinoids also modulate K^+^ channels. CB1 activation in neurons opens G-protein-coupled inward rectifier K^+^ channels (GIRKs) via Gβγ. If lung cancer cells express any GIRKs or other inward rectifiers (some do express Kir2.1 or Kir3.1 for maintaining resting potential), CB1/CB2 signaling might increase their activity, hyperpolarizing the cell. Hyperpolarization can have mixed effects: It can drive more Ca^2+^ entry through SOCs (thus promoting apoptosis if Ca^2+^ overloads), or it might stabilize the cell in a quiescent state by making depolarization more difficult. The net effect in a non-excitable cell context is likely an enhancement of Ca^2+^ store refill and perhaps reduced cell motility (extreme hyperpolarization tends to reduce lamellipodia dynamics). Aside from GIRKs, certain K2P (two-pore) channels that set resting potential can be modulated by cannabinoids. TREK-1, a K2P channel, is activated by polyunsaturated fatty acids and some cannabinoids indirectly; if lung cancer cells have TREK-1, cannabinoids could activate it to hyperpolarize the cell and induce apoptosis (hyperpolarization has been linked to initiation of apoptosis via facilitating cytochrome c release in some studies) [11,54,92]. On the other hand, we should note that cannabinoids might downregulate some K^+^ channels. For example, THC was reported to decrease expression of Kv2.1 in a glioma model, impacting viability. Perhaps in lung cancer, prolonged CB2 activation reduces KCa3.1 levels (since KCa3.1 is often upregulated by inflammatory cytokines and hypoxia, conditions that might be alleviated by cannabinoids’ anti-inflammatory effects).

There is limited data on the direct effects of cannabinoids on Nav channels in cancer. However, cannabinoids at high doses can have local anesthetic-like properties (THC and CBD can block Nav currents in neurons). If a lung cancer cell relies on Nav1.5 for invasiveness, cannabinoids might directly inhibit Nav1.5 currents, as CBD has been shown to reduce sodium current amplitudes in neuronal preparations. Moreover, through CB1/CB2 Gβγ, cannabinoids could activate signaling that leads to Nav channel internalization or reduced expression. Any reduction in Nav activity could impair the cell’s invasive machinery as discussed earlier (Nav-driven H^+^ extrusion, etc.) [93,94]. This is speculative, but a potential area where cannabinoids’ broad bioactivity could incidentally hamper metastatic processes.

#### 5.1.3. Chloride Channels and Others

Some cannabinoids can also modulate Cl^−^ channels (for instance, anandamide activates a calcium-dependent Cl^−^ current in certain cells). In lung cancer, Cl^−^ channels (like TMEM16A and CaCC) are important for cell volume regulation during migration [95,96]. If cannabinoids disturb Cl^−^ channel function (directly via channel binding or indirectly via Ca^2+^ changes), they might impede the cell’s ability to shrink and squeeze through the matrix, thus reducing invasiveness.

Cannabinoids exert a polyvalent influence on ion channels: elevating intracellular Ca^2+^ via TRP activation (causing cytotoxic stress), hyperpolarizing membranes via K^+^ channel activation (potentially leading to cell cycle arrest or augmented Ca^2+^ influx through certain pathways), and inhibiting excitatory ion fluxes via channel blockade (reducing pro-growth signals). The outcome is usually tipped toward growth inhibition and death of cancer cells. It is this broad-spectrum electrophysiological perturbation that can “reprogram” a lung cancer cell from a proliferative state to a stressed, dying state. Notably, these ionic effects happen concurrently with GPCR-mediated effects because some are initiated by the same receptor (CB1/CB2); for example, CB2 activation can both inhibit adenylyl cyclase (GPCR effect) and, through βγ, inhibit Ca^2+^ channels (ion channel effect). This coordination likely amplifies anti-tumor signaling (e.g., lowering cAMP while limiting Ca^2+^ influx needed for proliferation).

### 5.2. Biased Agonism of GPCRs by Cannabinoids

Biased agonism refers to the ability of different ligands for the same GPCR to differentially activate downstream pathways. A ligand might preferentially engage G proteins over β-arrestins or favor one G protein subtype over another, leading to distinct biological outcomes. Cannabinoids provide interesting examples of biased agonism at their receptors and possibly at other GPCRs relevant to lung cancer.

At CB1 receptors, THC and synthetic cannabinoids can exhibit bias. For instance, studies have found that THC is not a pure “neutral” agonist; it has a bias profile where it only weakly activates Gαi but more strongly recruits β-arrestin-1 and perhaps signals via Gαq. One report indicated THC’s signaling at CB1 in transfected cells was biased toward β-arrestin-1 and away from Gαi [97]. This might explain why THC can activate ERK/MAPK (which often requires β-arrestin scaffolding for sustained activation) without robustly inhibiting adenylyl cyclase in some systems. In contrast, synthetic agonists like WIN55,212-2 or CP55,940 often fully activate Gαi (strong cAMP inhibition) and have different arrestin recruitment profiles. What does this mean for cancer? If a cannabinoid agonist is G protein-biased at CB1/CB2, it might induce one set of outcomes (say, more PI3K/AKT activation initially), whereas an arrestin-biased agonist could cause another (like more ER stress and autophagy). It has been proposed that β-arrestin-biased CB1 agonists might be more effective at inducing cancer cell death because they provoke sustained ERK activation linked to the pro-apoptotic TRIB3/CHOP pathway, whereas G protein-biased agonists might produce transient signals that the cell can adapt to. Supporting this, a study in gastric cancer found that a CB1 agonist induced EMT inhibition via a β-arrestin-1 dependent pathway, independent of Gαi [7,43,98]. In lung cancer, leveraging bias could mean using ligands that maximize the pathways leading to apoptosis (e.g., p38, JNK, ER stress) while minimizing those that might promote survival (e.g., transient AKT activation).

At CB2 receptors, biased signaling is also documented. CB2 can couple to multiple Gα types (primarily Gi, but also reportedly Gs or G12 in certain contexts) and recruit β-arrestins. Different agonists have varying propensities to cause receptor internalization via β-arrestin. For example, the cannabinoid HU-308 (a CB2 agonist) might strongly activate Gi (cAMP inhibition) with minimal arrestin recruitment, whereas a structurally distinct agonist might do the opposite. An interesting study showed that two common human CB2 variants had different bias profiles: One was more arrestin-biased, potentially affecting how they modulate immune cell migration [99,100]. For cancer therapy, a Gi-biased CB2 agonist might be desired if one wants to primarily shut down cAMP and activate the AKT cascade, but wait, that sounds counterintuitive because activating AKT is usually pro-survival. However, AKT activation by CB2 is often transient; the long-term effect is that it can lead to feedback inhibition of the PI3K pathway or perhaps a switch to autophagy (as seen with some cannabinoids that initially activate AKT but then trigger ER stress). Alternatively, an arrestin-biased CB2 agonist might induce receptor internalization and possibly co-internalize CXCR4 (if heterodimerized), which is beneficial for reducing metastasis [52,63]. That same arrestin bias could scaffold p38 MAPK activation, leading to cancer cell senescence or apoptosis. Thus, bias engineering might allow dissection of CB2′s anti-tumor benefits from any immunosuppressive side effects (since Gi activation in immune cells by CB2 can limit their activity, whereas biasing away from Gi might preserve immune function while still affecting the tumor).

Beyond cannabinoid receptors, cannabinoids can bias heteromeric receptor signaling. For example, A2A-CB1 heteromers have been reported in the striatum; CBD was shown to allosterically modulate these A2A-CB1 complexes, altering β-arrestin recruitment. In the immune system, A2A-CB2 heteromers exist on neutrophils; CBD could bias their signaling, accelerating β-arrestin-mediated desensitization of chemotaxis. If similar heteromers exist in the lung cancer microenvironment (say on macrophages or endothelial cells), cannabinoids might preferentially tune those receptor signals. For instance, CBD might make A2A-CB2 heteromers signal in a way that promotes anti-tumor immunity instead of immunosuppression [57]. This is speculative but within the realm of bias–essentially, cannabinoids can bias receptor crosstalk.

Another facet of bias is as follows: ligand-specific receptor conformations. THC vs. endocannabinoids vs. synthetic agonists likely stabilize different CB1 conformations, leading to varied interactions with ion channels too. A conformation that strongly presents the Gβγ binding interface will robustly activate GIRKs and inhibit Ca^2+^ channels, whereas one that does not will favor other routes. Thus, THC might cause more K^+^ current bias (leading to hyperpolarization and perhaps a distinct outcome) compared to a synthetic ligand that causes less K^+^ current but more ERK activation. These nuanced differences could be exploited [101,102]. For instance, abnormal cannabidiol (abn-CBD) is a CBD isomer that does not activate CB1/CB2 but can activate endothelial receptors, and some speculate fthat it engages GPR55 or an “endothelial cannabinoid receptor”. Abn-CBD has been shown to cause NO release and vasodilation (Gs biased perhaps). In a tumor, a ligand like abn-CBD might target the vasculature differently than the tumor cells. Understanding each ligand’s bias profile allows tailoring combinations: e.g., combine a CB2 agonist that biases toward anti-migratory signaling with a CB1 agonist that biases toward pro-apoptotic signaling [103,104].

One concrete example of biased utility is as follows: A 2010 study found that a *CB2* agonist that did not recruit β-arrestin was effective in reducing ErbB2+ breast cancer growth via AKT inhibition. This suggests G protein signaling alone from CB2 was sufficient to impede AKT-driven tumors. On the contrary, for inducing cancer cell death via apoptosis, we might want a ligand that recruits β-arrestin to CB1 to fully trigger caspase cascades [7,105]. The biased agonism concept also ties into side effect management: CB1 β-arrestin pathways in neurons are linked to tolerance and some side effects, so a peripheral CB1 agonist for cancer that is Gi-biased might have less psychoactivity.

Cannabinoids as biased ligands means that we can potentially fine-tune which signaling pathways in a lung cancer cell are activated. By selecting or designing cannabinoids that favor, say, β-arrestin and p38 activation (to drive apoptosis) while avoiding too much Gi/AKT (which might transiently promote survival), we can maximize anti-cancer efficacy. This is an emerging area, and ongoing research is decoding the bias profiles of numerous synthetic cannabinoids and minor phytocannabinoids. Figure 4 conceptually shows how different cannabinoid ligands (THC vs. CBD vs. synthetic analogs) might push the CB1/CB2 signaling network in divergent directions (leading to cell survival vs. cell death outcomes).

Next, we integrate all these mechanistic insights to explain how cannabinoids “rewire” the signaling networks of lung cancer cells, shifting them from an oncogenic state toward a state of vulnerability (characterized by inhibited growth, induction of apoptosis, reduced motility, and greater sensitivity to other therapies).

## 6. Rewiring of Oncogenic Signaling by Cannabinoids: Impact on EGFR, PI3K-AKT, and Other Pathways

**Figure 5:**
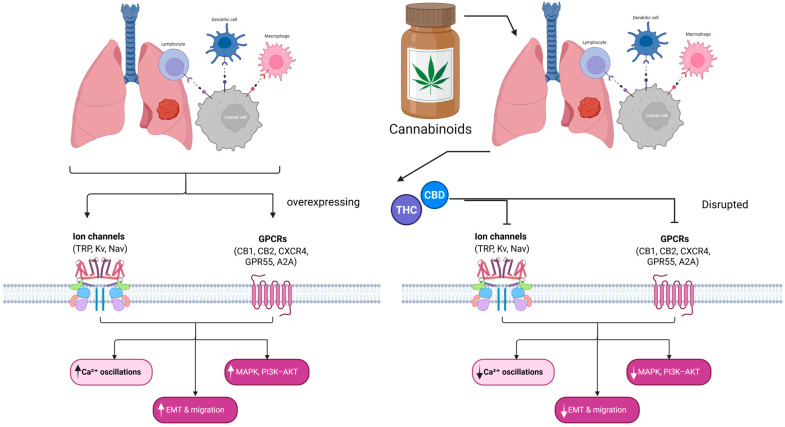
Cannabinoids as rewiring agents of oncogenic electrophysiology in lung cancer. Cannabinoids disrupt GPCR–ion channel crosstalk by collapsing Ca^2+^ microdomains and altering membrane signaling architecture. This electrophysiological rewiring attenuates EGFR and PI3K–AKT signaling, suppressing proliferation, migration, and therapy resistance in lung cancer cells. This figure was created via Biorender Premium License by Fahrul Nurkolis.

One of the remarkable effects of cannabinoid treatment in cancer cells is the broad reprogramming of intracellular signaling networks. In lung cancer, two critical pathways often hyperactivated are the EGFR (ErbB) signaling cascade and the PI3K-AKT-mTOR pathway. Together, these drive proliferation, survival, and metabolic adaptation. Cannabinoids have been shown to intersect with and modulate these pathways at multiple nodes, thereby “rewiring” the flow of information that dictates cell fate (Figure 5). Additionally, cannabinoids influence the MAPK pathways (ERK, JNK, and p38), the NF-κB pathway, and the apoptotic machinery.

### 6.1. EGFR Signaling

Epidermal growth factor receptor (EGFR) is frequently overexpressed or constitutively active in NSCLC, making it a prime driver of tumor growth and a key therapeutic target (with EGFR-TKIs in the clinic). Cannabinoids appear to downregulate and antagonize EGFR signaling in lung cancer cells. As mentioned earlier, THC and CBD caused a reduction in EGFR mRNA and protein levels in multiple NSCLC cell lines. This effect was observed alongside upregulation of E-cadherin (an epithelial marker) and downregulation of mesenchymal markers, indicating that EGFR-driven EMT was being reversed [9,106]. The mechanism may involve cannabinoid-induced downregulation of transcription factors that promote EGFR (such as c-Jun or hypoxia-inducible factors) or even epigenetic modifications. There is evidence in other cancers that activation of CB1/CB2 leads to suppression of the EGFR ligand system: For instance, in breast cancer cells, a CB2 agonist reduced the production of heregulin (an EGFR/HER3 ligand) and hampered EGFR pathway activity [7]. In lung cancer, cannabinoids might increase the expression of EGFR negative regulators. One candidate is LRIG1, a feedback inhibitor of EGFR; if cannabinoids upregulate LRIG1 or similar EGFR ubiquitin ligases, they could target EGFR for degradation. Another route is via suppression of IGF-1R signaling; recall TRPV1 knockdown inhibited IGF-1R in NSCLC, and cannabinoids can reduce TRPV1′s pro-IGF signaling [14,107,108]. Lower IGF levels can, in turn, reduce EGFR cross-talk (EGFR and IGF-1R often co-sustain AKT activity). Importantly, EGFR transactivation by GPCRs is curbed by cannabinoids: Many GPCRs (like CXCR4, LPA receptors) transactivate EGFR via metalloprotease cleavage of pro-EGF ligands. CB2 activation interferes with CXCR4, thus likely reducing CXCL12-induced EGFR activation. Also, if cannabinoids reduce matrix metalloproteinase activity (some studies show CBD lowers MMP-9 and ADAM17 expression), the shedding of EGF family ligands is inhibited, preventing EGFR activation [7]. Additionally, cannabinoids induce ER stress in tumor cells, which can lead to the upregulation of protein tyrosine phosphatases (like PTP1B) that dephosphorylate EGFR, thus attenuating its signal. Summarily, cannabinoids dampen EGFR signaling at multiple levels: transcriptional downregulation, decreased ligand availability, increased receptor turnover, and enhanced dephosphorylation. The consequence is a re-routing of cell signaling away from the EGFR-RAS-ERK axis that drives proliferation toward alternative pathways (like stress kinase JNK or p38, which drive apoptosis). In EGFR-driven lung cancers, such as those with activating EGFR mutations, cannabinoids might provide a complementary approach: by reducing EGFR expression and downstream output, they could resensitize tumors to EGFR inhibitors or overcome certain resistance mechanisms (e.g., in resistant cells that upregulate alternate pathways, cannabinoids pressing EGFR down further can tip the balance to cell death).

### 6.2. PI3K-AKT-mTOR Pathway

This pathway is a central regulator of survival and metabolism in lung cancer, often activated by EGFR or mutant KRAS, and associated with chemotherapy and TKI resistance. Cannabinoids have consistently been shown to inactivate PI3K/AKT/mTOR signaling in cancer cells. For instance, in lung cancer, AEA and synthetic agonists decreased phosphorylated AKT levels and mTOR activity, correlating with induction of autophagy and apoptosis [7,109]. Jeon et al. (2025) found that combining CBD with etoposide led to robust dephosphorylation of AKT and mTOR, thereby disabling a key survival pathway [109]. They noted that this did not rely on classical cannabinoid receptors or TRP channels, suggesting that CBD might directly target upstream regulators of AKT (for example, CBD can activate AMPK, which in turn activates TSC2, the inhibitor of mTORC1). In many cannabinoid studies, an increase in the stress protein TRIB3 is observed; TRIB3 binds AKT and prevents its activation. THC in glioma models induced TRIB3 via the p8/CHOP ER stress pathway, leading to sustained AKT inhibition and apoptosis. It is likely that a similar mechanism occurs in lung cancer cells: Cannabinoids cause ER stress (perhaps due to disruption of ER Ca^2+^ homeostasis via TRP channels and due to ceramide accumulation), triggering CHOP and TRIB3 expression, which then bind to AKT and block its phosphorylation. With AKT inhibited, its downstream targets that promote survival (such as MDM2, GSK-3β, NF-κB, and mTORC1) are inactivated. Indeed, cannabinoid-treated lung tumors show reduced phosphorylation of ribosomal S6 protein and 4EBP1, hallmarks of mTORC1 suppression, consistent with an anti-proliferative, pro-apoptotic shift [7]. Another aspect is that cannabinoids often increase AMPK activity (as mentioned, possibly via CaMKKβ due to elevated Ca^2+^ or via ROS). Activated AMPK directly phosphorylates TSC2 and Raptor, leading to mTORC1 inhibition and induction of autophagy. Autophagy can be a double-edged sword, but in many cannabinoid studies, it appears to be a mechanism of cell death (termed autophagic cell death), especially in NSCLC, where p53 status can determine whether autophagy becomes cytotoxic. Jeon et al. (2025) noted that CBD + etoposide upregulated autophagy genes and that this response was p53-dependent interestingly, if p53 is intact, cannabinoids pushing cells into autophagy leads to self-digestion and death; if p53 is mutated, cells might escape this fate, which suggests a possible patient stratification (cannabinoid therapies might work better in p53 wild-type tumors, or combining with agents that restore p53 could synergize with cannabinoids) [109]. Importantly, the cannabinoid-induced AKT/mTOR blockade enhances sensitivity to other treatments. For example, radiation and many chemotherapies rely on active apoptosis pathways, which are often blocked by AKT. By removing that block, cannabinoids could amplify DNA-damage-induced cell death. There is evidence that cannabinoids synergize with EGFR-TKIs partly through this route: EGFR inhibition alone only partially reduces AKT activity (due to feedback loops), but adding a cannabinoid (which hits AKT from a different angle) yields a more complete shutdown of PI3K/AKT, driving cells into apoptosis [2,110,111].

### 6.3. MAPK Pathways

Cannabinoids modulate the MAPK cascades (ERK1/2, JNK, p38) in lung cancer. Generally, ERK1/2 is a pathway often promoting proliferation, initially activated by cannabinoids (via CB1/2 or TRP-induced Ca^2+^), but this activation can either be transient or sustained in a way that triggers pro-death signals. Some studies show a biphasic ERK response: a peak of ERK phosphorylation early (30–60 min) that might help induce autophagy or cell cycle arrest, followed by a return to baseline or below baseline as upstream drivers (EGFR, AKT) are shut off. Sustained ERK via β-arrestin scaffolds may push cells to senescence or differentiation. JNK and p38 are stress-activated kinases that often promote apoptosis. Cannabinoids have been shown to activate p38 MAPK in lung cancer cells, contributing to apoptosis via p38-mediated upregulation of death receptors and Bax. For instance, WIN55,212-2 increased phosphorylated p38 in NSCLC, and pharmacologic inhibition of p38 partially rescued cells from apoptosis, indicating p38 was an executioner in cannabinoid-induced cell death. Similarly, JNK can be activated by ceramide accumulation due to cannabinoid treatment; JNK, in turn, activates pro-apoptotic proteins (Bim) and inactivates Bcl-2. In a study on lung cancer cell migration, CBD plus another cannabinoid was found to reduce migration through p38 activation and AKT inhibition, which led to decreased secretion of MMP-2/9 and downregulation of focal adhesion kinase (FAK) [7,112,113]. In summary, cannabinoids tilt the balance of MAPK signaling from growth (ERK) toward stress (JNK/p38), reinforcing pathways that culminate in cell death.

### 6.4. NF-κB Pathway

NF-κB is often constitutively active in lung cancer, driving expression of survival genes (e.g., Bcl-xL, XIAP) and inflammatory factors (IL-6, IL-8). Cannabinoids can suppress NF-κB signaling. Through CB2 Gi, cannabinoids can inhibit adenylyl cyclase and PKA, and since PKA can phosphorylate IκB (promoting NF-κB activation), reduced PKA might stabilize IκB, keeping NF-κB sequestered. More directly, cannabinoid-induced ceramide has been shown to prevent NF-κB p65 nuclear translocation in some cancer cells. In lung adenocarcinoma models, treatment with THC or CBD led to decreased nuclear NF-κB levels and lower IL-8 production. Since IL-8 is a pro-survival and pro-angiogenic chemokine, its downregulation by cannabinoids (observed in TRPV1 studies and by direct CB receptor activation) contributes to a less aggressive phenotype [3,114]. Additionally, by inhibiting NF-κB, cannabinoids remove a block on apoptosis (many NSCLCs rely on NF-κB to keep apoptotic pathways off, via upregulating inhibitors of apoptosis proteins). Indeed, cannabinoid treatment in NSCLC was associated with reduced expression of IAPs and FLIP, tipping the balance toward caspase activation upon any death stimulus.

### 6.5. Apoptotic Pathways

The culmination of signaling changes results in activation of the intrinsic and extrinsic apoptosis pathways. Cannabinoids elevate pro-apoptotic factors: E.g., they increase the Bax/Bak ratio to Bcl-2/Bcl-xL (through p53 activation or NF-κB inhibition), cause mitochondrial membrane permeabilization (through Ca^2+^ and ROS), and activate caspase-3, -7, and -9. They can also increase death receptor DR5 (via CHOP upregulation in ER stress), making cells more susceptible to extrinsic triggers. Several studies confirmed caspase-dependent apoptosis in lung cancer cells upon cannabinoid exposure. For example, Misri et al. showed that CBD treatment led to cleavage of caspase-3 and PARP in cisplatin-resistant cells, indicating execution of apoptosis [10]. Meanwhile, autophagy acts concurrently; often, blocking autophagy in these settings enhances apoptosis, suggesting that the two are linked in a death continuum (autophagy might be helping lead to apoptosis by degrading anti-apoptotic proteins or causing lethal atrophy).

### 6.6. Metastatic and Invasive Signaling

Rewiring by cannabinoids also results in *anti-migratory and anti-metastatic* outcomes. By inhibiting EGFR and AKT, cannabinoids reduce cell motility signals (EGFR→Rac1 and AKT→PAK1 pathways are needed for lamellipodia; their inhibition leads to collapse of these structures). Moreover, cannabinoids reduce matrix metalloproteinases (MMP-2 and MMP-9), likely via downregulating NF-κB and AP-1 transcription factors [7]. For example, a combination of CBD and cannabidivarin was found to upregulate TIMP-1 (tissue inhibitor of MMPs) in colon cancer models, which would impede metastasis. In lung cancer, as noted, THC/CBD restored *E-cadherin* and reduced *vimentin*, essentially reversing EMT [7,9]. This mesenchymal-to-epithelial transition makes cells less invasive and more sensitive to anoikis (detachment-induced cell death). Additionally, through CB2, cannabinoids interfere with chemotactic signaling (CXCR4/CXCL12 axis), meaning that tumor cells are less responsive to metastatic niches cues [64]. In vivo, cannabinoids have been shown to reduce the number and size of lung metastases in models of other cancers (e.g., breast cancer metastasis to lung was reduced by THC treatment, partly by lowering CXCR4 and ID1 expression) [7]. Yoon and Grimsey (2025) demonstrated that a cannabis extract significantly decreased pulmonary metastases of colorectal cancer cells in mice, correlating with AMPK activation and MAPK inhibition in the tumor cells [48,100]. This highlights that beyond primary tumor growth, cannabinoids rewire signaling to create an environment hostile to metastatic colonization: they promote tumor cell dormancy or death rather than active invasion.

Cannabinoids affect the multi-target “rewiring” of lung cancer cell signaling—turning off major growth and survival switches (EGFR, PI3K/AKT, and NF-κB) and turning on stress and death pathways (p38/JNK, CHOP-TRIB3, caspases, autophagy). This comprehensive shift underlies the observed functional consequences. In addition to their effects on oncogenic signaling pathways, cannabinoid-driven rewiring may also influence the response of lung cancer cells to radiotherapy.

Radiotherapy remains a cornerstone in the management of lung cancer, exerting its therapeutic effect primarily through the induction of DNA damage and ROS-mediated cytotoxicity. Emerging evidence suggests that GPCR and ion channel signaling networks critically influence radiation sensitivity by regulating intracellular Ca^2+^ dynamics, redox balance, and DNA damage responses [115]. In this context, cannabinoid-driven modulation of these pathways may significantly alter the radiation response of lung cancer cells. For instance, activation of TRP channels such as TRPV2 by cannabinoids can enhance Ca^2+^ influx and oxidative stress, potentially amplifying radiation-induced cytotoxicity [116]. Concurrently, suppression of pro-survival GPCR signaling pathways, including PI3K–AKT–mTOR and EGFR cascades, may reduce cellular capacity for DNA repair and survival following irradiation [117].

Cannabinoids may also influence key determinants of radioresistance, including antioxidant defense systems and tumor microenvironmental factors. Notably, downregulation of NRF2 signaling by cannabinoids has been associated with impaired cellular antioxidant responses, leading to accumulation of ROS and increased susceptibility to radiation-induced damage [118]. In parallel, modulation of ion channel activity, particularly potassium and calcium channels, can disrupt mitochondrial function and membrane potential, further sensitizing cancer cells to oxidative stress. Additionally, cannabinoid-mediated inhibition of NF-κB signaling may attenuate radiation-induced pro-survival and inflammatory responses, thereby limiting adaptive resistance mechanisms that often undermine radiotherapy efficacy [119].

Beyond direct tumor cell effects, cannabinoid-driven rewiring of GPCR–ion channel signaling may also impact the tumor microenvironment and vascular responses to radiation. Cannabinoids have been reported to modulate angiogenesis and immune cell infiltration, both of which are critical determinants of radiotherapy outcomes. For example, reduced VEGF signaling and normalization of tumor vasculature could improve oxygenation, thereby enhancing radiosensitivity, while alterations in immune signaling may influence radiation-induced immunogenic cell death [120,121]. Collectively, these observations suggest that cannabinoids hold potential as radiosensitizing agents through multi-level modulation of signaling networks. However, further mechanistic and in vivo studies are required to define optimal combinations, dosing strategies, and potential context-dependent effects in lung cancer radiotherapy.

## 7. Functional Consequences of Cannabinoid-Induced Signaling Rewiring

By altering the signaling landscape in lung cancer cells, cannabinoids engender a series of anti-tumorigenic functional outcomes. The key phenotypic changes observed, and their mechanistic underpinnings, are:

### 7.1. Induction of Cancer Cell Death and Growth Arrest

As detailed, cannabinoids trigger programmed cell death in lung cancer cells. Morphologically and biochemically, treated cells show chromatin condensation, DNA fragmentation, phosphatidylserine externalization, and cleavage of caspases/PARP [10]. Apoptosis is often both intrinsic (mitochondrial) and extrinsic in nature. Intrinsically, mitochondrial outer membrane permeabilization (MOMP) occurs due to high calcium (activating calpains and Bax) and ROS, releasing cytochrome c and activating caspase-9 [2,122]. Extrinsically, as mentioned, cannabinoids upregulate death receptors like DR5 via CHOP, so in the presence of death ligands (or through autocrine TNF-α, which some cancer cells produce), caspase-8 gets activated. Apoptosis is a major reason for tumor growth inhibition observed in xenograft models treated with cannabinoids: E.g., in mice with NSCLC xenografts, THC administration led to significant tumor necrosis and increased TUNEL-positive apoptotic cells compared to controls [7]. Interestingly, apoptosis can also occur in the tumor endothelium with cannabinoids, impairing angiogenesis (THC was shown to induce apoptosis of tumor endothelial cells via CB2, cutting off blood supply to lung tumors in a rat model).

Many cannabinoids stimulate autophagy in lung cancer cells, evidenced by LC3-II accumulation and autophagosome formation. Autophagy can serve as a cell survival mechanism under stress; however, when excessively induced or when apoptosis is simultaneously blocked, it can lead to autophagic cell death. In NSCLC, autophagy induced by CBD (especially in combination with DNA-damaging agents) overwhelmed the cells, contributing to cell death beyond what apoptosis alone could achieve. Notably, in p53 wild-type settings, autophagy and apoptosis can cooperate (p53 upregulates pro-apoptotic genes and also some autophagy genes). In p53 mutant settings, cannabinoids still induced autophagy, but it might have been cytostatic rather than cytotoxic [109,112,123]. Nonetheless, the functional outcome is that cell proliferation halts and cell viability plummets due to self-cannibalization. Autophagy also has an immunogenic aspect; if it leads to the release of ATP or HMGB1 from dying cells, it can trigger anti-tumor immune responses (immunogenic cell death). There is emerging evidence that THC and CBD can cause immunogenic cell death in some cancer cells, making them more visible to immune cells; this could be advantageous if properly harnessed.

Before cells die, they often undergo cell cycle blockade. Cannabinoids cause accumulation of lung cancer cells in the G0/G1 phase or sometimes in G2/M (some reports of THC causing a mitotic block via cyclin B1 reduction) [22]. The G1 arrest is linked to increased p27^Kip1^ (due to AKT inactivation and FOXO3a activation) and decreased cyclin D and E levels [9]. For example, in A549 cells, a CB1 agonist (ACEA) reduced cyclin D1 expression, slowing progression through the restriction point. G2/M arrest, seen in a few studies, was associated with CHK1/CHK2 activation from DNA damage and reduced Cdc2 activity. The net effect is fewer cancer cells entering mitosis, thus fewer cells dividing. In vivo, tumors treated with cannabinoids often show reduced proliferation indices (Ki-67 staining drops significantly) [3]. This cytostatic effect complements the cytotoxic effect (apoptosis) by halting tumor expansion.

### 7.2. Suppression of Tumor Progression and Microenvironmental Remodeling

Functionally, cannabinoids impair the ability of lung cancer cells to move and invade through the extracellular matrix. In wound-healing assays, both THC and CBD significantly reduced the migration of NSCLC cells (A549, H460, H1792) in response to EGF. The combination of THC + CBD had a greater effect than either alone, nearly abrogating scratch closure over 24–48 h [9]. Mechanistically, this mirrors the signaling changes: reduced EGFR and AKT signaling leads to lower Rac1 activity (cells cannot form lamellipodia effectively), and increased E-cadherin makes cells more epithelial and less motile. In Boyden chamber invasion assays, cannabinoid-treated lung cancer cells show diminished ability to penetrate Matrigel, correlating with decreased MMP-2/9 gelatinase activity in conditioned media [7,124]. The upregulation of TIMP-1 by cannabinoids noted earlier also directly inhibits MMPs, a crucial step for invasion. Furthermore, by reversing EMT, cannabinoids increase cell–cell adhesion (E-cadherin-mediated), which inherently suppresses individual cell migration and metastasis. For instance, Milian et al. observed that after THC/CBD treatment, NSCLC cells maintained stronger adherens junctions (visualized by membrane E-cadherin staining) and moved collectively much more slowly [9]. In vivo metastasis models lend credence: lung cancer cells pre-treated with cannabinoids formed fewer metastatic nodules when injected into mice, implying that their invasive and colonization capacity was crippled.

Although not the main focus of this review, it is worth noting as a functional consequence that cannabinoids can also disrupt tumor angiogenesis. Lung tumors rely on neovascularization; cannabinoids, via CB1/CB2 on endothelial cells, inhibit pro-angiogenic signaling (e.g., VEGF is downregulated via HIF-1α suppression when AKT/mTOR is inhibited). Research has shown that cannabinoids can reduce microvessel density in xenograft tumors [7]. This stems from both direct effects (CB2 activation on endothelial cells triggers apoptosis) and indirect effects (less VEGF and IL-8 from tumor cells). For instance, in a K-ras^LA1^ lung cancer mouse model, oral CBD reduced the expression of angiogenic factors and significantly cut down the formation of new blood vessels around tumor foci [48]. This additional anti-angiogenic effect means that the tumor’s blood supply is choked off, enhancing hypoxia and tumor cell death, which is ironically a positive feedback since the cannabinoids leverage stress to kill cells (via CHOP induction, etc.).

A complex consequence is on the immune system. Cannabinoids are known immunomodulators that are generally anti-inflammatory. In the context of cancer, this could be a double-edged sword: reduced inflammation might hamper anti-tumor immunity. However, in some settings like lung cancer, the immune environment is often immunosuppressive (rich in TAMs, MDSCs, and TreGs); interestingly, cannabinoid signaling can alter the balance of immune cell phenotypes. As noted, TRPV1 knockdown increased infiltration of cytotoxic T cells in tumors [14]. While TRPV1 is not immune cell-specific, it modulated tumor GABA production and macrophage polarization. Likewise, CB2 activation can skew macrophages from M2 (tumor-promoting) to M1 (tumoricidal), evidence: CB2 agonist JWH-015 prevented IL-4-induced M2 polarization in macrophages, which in lung cancer could translate to less support for tumor growth [7,125]. Additionally, by downregulating CXCL12/CXCR4, cannabinoids reduce recruitment of immunosuppressive cells to the tumor site and may trap tumor cells in the primary site (less metastasis). Some preclinical studies combining cannabinoids with immune checkpoint blockers (e.g., anti-PD-1) indicate that there is no negative interference and possibly an additive effect, but this area is still under investigation. In summary, while cannabinoids may dampen some inflammatory components, the net effect might still favor tumor control if it reduces pro-tumor inflammation and does not severely impede cytotoxic lymphocytes. Given that NSCLC patients often have a chronic inflammatory microenvironment that actually supports tumor growth (via NF-κB, IL-6, etc.), cannabinoids’ anti-inflammatory action could alleviate tumor-promoting inflammation while direct tumor cell-killing proceeds.

### 7.3. Overcoming Therapy Resistance

Perhaps one of the most clinically relevant functional outcomes is that cannabinoids can sensitize lung cancer cells to other therapies or overcome resistance mechanisms. This has touched on synergy with EGFR inhibitors and chemotherapy. NRF2 is a key transcription factor that regulates defenses against oxidative stress and reactive oxygen species [126]. To elaborate, cisplatin resistance in NSCLC is frequently associated with increased NRF2-driven antioxidant defenses and drug efflux pumps. CBD in Misri et al.’s study reduced NRF2 levels, and ROS was allowed to accumulate, thereby re-sensitizing cells to cisplatin [10]. It also downregulated the expression of multidrug resistance genes (like ABCA5 in the TRPV1 context and possibly others like ABCG2 in general). Similarly, erlotinib resistance often involves MET pathway activation or epithelial–mesenchymal transition; cannabinoids by restoring epithelial markers and suppressing alternate growth pathways (like MET via SOCS induction perhaps) could counteract TKI resistance. There is emerging evidence that a derivative of THC (Δ^8^ THC) combined with Osimertinib (3rd-gen EGFR TKI) had enhanced efficacy in an EGFR-mutant NSCLC model, putatively because THC induced autophagy that circumvented a resistance pathway to Osimertinib (since some resistant cells depend on autophagy avoidance). Additionally, radiation therapy relies on ROS and DNA damage; cannabinoids elevate ROS in tumors (via TRPV2 and mitochondrial effects), which might act as radiosensitizers [10,127]. In fact, a concept in the Frontiers article was “nanoparticle drones” delivering cannabinoids with radiosensitizers to lung tumors to increase radiation killing [61]. Moreover, by causing cell cycle arrest in G1, cannabinoids can synchronize cells or prevent repair during radiation, making them more prone to die when DNA damage occurs.

The functional changes increased cancer cell death, reduced migration/invasion, decreased angiogenesis, and potentially improved responses to other treatments, illustrating a comprehensive anti-cancer effect profile for cannabinoids in lung cancer. Importantly, these outcomes are achieved through multi-target engagement, which might reduce the likelihood of resistance development (the tumor would have to simultaneously adapt to multiple signaling disruptions).

## 8. Future Therapeutic Strategies

The growing body of evidence reviewed herein suggests that cannabinoid-driven rewiring of cell signaling offers a multifaceted assault on lung cancer. Moving forward, several strategies could translate these insights into improved therapies.

### 8.1. Selective Cannabinoids and Synthetic Analogues

One approach is to develop or utilize cannabinoids that selectively target tumor-expressed receptors with minimal psychoactive effects. For example, CB2-selective agonists (like JWH-133, HU-308) could be employed to harness anti-tumor immunity and direct tumor apoptosis without engaging CB1 in the brain (thus avoiding psychotropic side effects). Clinical-grade CB2 agonists are under development for inflammation; repurposing them for lung cancer (especially metastatic or chemoresistant cases) is plausible. Another angle is peripherally restricted CB1 agonist compounds that activate CB1 (which is present in lung tumors) but do not cross the blood–brain barrier. Such compounds could maximize CB1′s pro-apoptotic effects in tumors while sparing patients the CNS effects of THC. Additionally, biased agonists can be leveraged: for instance, a CB1 agonist biased toward β-arrestin might induce greater apoptosis; a CB2 agonist biased away from Gi (to avoid immune suppression) but still causing tumor cell death would be valuable. High-throughput screening for cannabinoid analogues with these bias profiles is an ongoing area. Non-classical cannabinoids (e.g., CBD analogues, or compounds targeting GPR55 or TRPV channels) could also be part of a cocktail; CBD itself is appealing as an adjunct therapy given its safety profile and multi-target nature (it hits TRPV2, GPR55, PPARγ, etc.). One proposed approach is a combination of multiple minor cannabinoids: For example, a recent study showed combining CBD with cannabidivarin (CBDV) enhanced downregulation of CXCR4 and further reduced colon cancer cell migration [64]. Translating to lung cancer, using a combination like CBD + CBDV or THC + CBD in a defined ratio might produce synergistic anti-tumor effects via complementary signaling effects (the so-called “entourage effect” of cannabinoids).

### 8.2. Combining Cannabinoids with Conventional Therapies

Given cannabinoids’ ability to sensitize cancer cells, rational combination regimens could be designed. EGFR-TKIs + Cannabinoids: In EGFR-mutant NSCLC patients, adding a cannabinoid (e.g., a non-psychoactive CB2 agonist or CBD) might delay or overcome TKI resistance. The combination could target both the kinase signaling (TKI) and the ionic/GPCR network (cannabinoid), making it harder for tumor cells to adapt. Preclinical support comes from the KCa3.1 inhibitor + erlotinib study, which is analogous in concept [2]. Chemotherapy + Cannabinoids, for example, CBD + Platinum Doublets: CBD could protect normal tissues from radiation/chemo damage by reducing inflammation (as seen in a study where CBD protected lung endothelial cells from irradiation oxidative damage) while simultaneously making tumor cells more chemosensitive [128]. A clinical trial in the UK (2019) combined CBD with chemotherapy in pancreatic cancer patients and reported improved survival, fueling interest in similar trials for lung cancer. Immunotherapy + Cannabinoids: This is more complex due to cannabinoids’ immunomodulation. However, preliminary data suggest that cannabinoids do not blunt the efficacy of checkpoint inhibitors and may help with immune-related adverse events by mitigating cytokine storms. A careful dosing schedule (e.g., using cannabinoids during certain intervals to not continuously suppress immune activation) might allow synergy, for instance, using CB2 agonists to reduce MDSCs and Treg infiltration while unleashing T cells via PD-1 blockade. There is evidence that CB2 activation elevates ICAM-1 on tumor cells, potentially making them more susceptible to T cell attachment and killing; this could synergize with immunotherapy [7].

### 8.3. Nanocarrier Delivery and Tumor Targeting

To maximize tumor-specific effects and minimize systemic exposure, nanotechnology-based delivery of cannabinoids is promising. Inhalation methods (nebulizers for CBD/THC aerosols) could directly target lung tumors with high local concentration and low systemic levels. Nanoparticles (NPs) can be engineered to carry cannabinoids and be functionalized with tumor-homing peptides (like RGD, which targets integrins on lung tumor endothelium). Gold nanoparticles loaded with THC or CBD could also act as radiosensitizers, as gold NPs themselves enhance radiation damage [61]. There is also work on exosome-based delivery of cannabinoids: Exosomes can naturally target tumors (especially if derived from targeted cells) and were shown to deliver CBD effectively to reduce lung tumor growth in a mouse model [129]. Another concept is encapsulating cannabinoids in liposomes or polymeric NPs to improve their bioavailability and tumor uptake. Because cannabinoids are lipophilic, NPs help solubilize and protect them until they reach the tumor. Iron oxide nanoparticles bound to cannabinoids have even been proposed to combine magnetic hyperthermia with cannabinoid therapy, a dual modality where magnetic NPs heat up the tumor on an alternating magnetic field while releasing cannabinoids to kill heat-stressed cells [130].

### 8.4. Biomarker and Patient Stratification

As we translate this to the clinic, it will be important to identify which patients could benefit most from cannabinoid adjunct therapy. Potential Biomarkers: *CB2*-high tumors (by IHC or PET imaging with CB2 ligands) might respond better to CB2 agonists; *TRPV2*-high tumors (e.g., by mRNA profiling) might be particularly susceptible to CBD therapy [10]; *EGFR-TKI*-resistant tumors with certain bypass tracks (like IL-8/NF-κB-driven) might respond to CBD because it cuts IL-8 [3]. Also, *p53* status could be a factor; if a tumor is p53 wild-type, it might undergo cannabinoid-induced apoptosis more readily via p53 pathways; if p53-null, a higher dose or combination may be needed to rely on p53-independent killing (like via ER stress overwhelming threshold). Another stratifier is immune contexture: patients with low T cell infiltration (cold tumors) might not lose much by adding a cannabinoid and could gain them by conversion of macrophages to M1, whereas patients with very robust pre-existing T cells (hot tumors) must ensure that the cannabinoid does not stifle those T cells.

Sex-specific differences in cannabinoid signaling and ion channel regulation represent an important but underexplored dimension in lung cancer biology. While emerging evidence in other systems suggests that sex hormones can modulate endocannabinoid tone, GPCR signaling, and ion channel expression, direct comparative studies in male versus female lung cancer models remain limited. Future investigations addressing sex-dependent variability may provide important insights for precision therapeutic strategies. Ultimately, a personalized approach measuring cannabinoid receptor levels, TRP channel expression, and tumor immune profile could guide the inclusion of cannabinoid-based therapy.

### 8.5. Clinical Trials and Safety

Encouragingly, cannabinoids (especially CBD) are relatively well-tolerated. Sativex (an oromucosal spray of THC/CBD) has been tested in cancer pain and showed manageable side effects. In the context of lung cancer, quality of life could also improve (pain relief, appetite stimulation by THC, anti-nausea, etc.), which is a beneficial side effect. However, we must caution the risk of immunosuppression by high-dose THC in patients; careful dosing or focusing on non-psychoactive, non-immunosuppressive components is key. Clinical trials like the ongoing study combining CBD with Temozolomide in glioblastoma or with chemo in pancreas are paving the way; similar trials in lung cancer (perhaps CBD with pembrolizumab, or THC/CBD with EGFR-TKI) should be initiated. Given that both THC and CBD are now available in various formulations (and CBD is even FDA-approved for epilepsy), repurposing them could be done relatively quickly in early-phase trials.

## 9. Conclusions

In conclusion, mounting evidence indicates that cannabinoids can significantly impact lung cancer signaling networks and tumor behavior. By acting on G protein-coupled receptors and ion channels, cannabinoids reprogram key oncogenic pathways by suppressing regulators of proliferation and survival while promoting apoptotic and differentiation-associated signaling. This polypharmacological profile, spanning calcium dynamics, kinase signaling cascades, and gene expression, represents a promising therapeutic avenue. Importantly, preclinical evidence suggests that cannabinoids may act synergistically with epidermal growth factor receptor inhibitors by attenuating compensatory pathways such as the phosphoinositide 3-kinase–protein kinase B–mechanistic target of rapamycin and mitogen-activated protein kinase signaling, thereby enhancing tumor cell apoptosis, reducing metastatic potential, and potentially improving anti-tumor immune responses while minimizing toxicity through targeted delivery strategies. Beyond lung cancer, the oncogenic roles of GPCRs and ion channel families, including TRP channels, potassium channels, voltage-gated sodium channels, and cannabinoid-responsive receptors, are increasingly recognized across multiple malignancies such as breast, glioma, colorectal, and pancreatic cancers. In these contexts, similar mechanisms involving Ca^2+^ signaling, membrane potential regulation, and GPCR-mediated pathway modulation converge to drive proliferation, invasion, and therapy resistance. Notably, cannabinoid-mediated modulation of these signaling networks has demonstrated anti-tumor activity across diverse cancer models, reinforcing the concept that GPCR–ion channel crosstalk represents a conserved and targetable vulnerability in cancer biology. These cross-cancer insights further support the translational potential of cannabinoid-based strategies as broadly applicable modulators of oncogenic signaling.

While challenges remain (optimal dosing, patient selection, and regulatory hurdles), the insight that can simultaneously target GPCRs and ion channels to cripple lung cancer is a paradigm shift. The convergence of cancer signaling biology with cannabinoid pharmacology opens up exciting possibilities for combination treatments that might tackle tumor resistance and recurrence. In summary, cannabinoid-driven modulation of GPCR and ion channel signaling represents a promising multi-pronged strategy against lung cancer, warranting further investigation and translation into clinical trials. If successful, such approaches could complement existing therapies and improve outcomes for patients with this devastating disease.

## Data Availability

No data were produced from this study; all data are contained in this article and the published papers listed in the references.
